# Hypoxia-induced lnc-IRP drives oxaliplatin resistance in colorectal cancer through IRP2 sequestration and iron metabolism reprogramming

**DOI:** 10.1186/s12967-026-08440-3

**Published:** 2026-06-22

**Authors:** Haiou Yang, Xinyi Wang, Shuang Li, Mingqing Zhang, Zhengyang Zhou, Jiayu Yang, Xipeng Zhang, Ming Gao, Haiyang Zhang

**Affiliations:** 1 Cancer Center, Shanxi Bethune Hospital, Shanxi Academy of Medical Sciences, Tongji Shanxi Hospital, Third Hospital of Shanxi Medical University, Taiyuan, Shanxi 030032 China; 2 Tianjin Union Medical Center, Tianjin Key Laboratory of General Surgery in Construction, Tianjin Institute of Coloproctology, The First Affiliated Hospital of Nankai University, Tianjin, 300121 China; 3 Department of Gastroenterology, Tianjin First Central Hospital, Tianjin, 300192 China; 4 National Clinical Research Center for Cancer, Key Laboratory of Cancer Prevention and Therapy, Tianjin Medical University Cancer Institute and Hospital, Tianjin, 300202 China; 5 Shandong Provincial Hospital Affiliated to Shandong First Medical University, Jinan, 250021 China

**Keywords:** Colorectal cancer (CRC), Hypoxia, Iron metabolism, Non-coding RNA, Oxaliplatin

## Abstract

**Background:**

Hypoxic microenvironment is an important characteristic of solid tumors. A series of adaptations associated with hypoxia occur in cancer cells, including reprogramming of multiple metabolic pathways. Gastrointestinal bleeding is a common clinical symptom of CRC, resulting in anemia and iron deficiency. CRC patients have unbalanced iron homeostasis due to blood loss, chronic inflammation, etc. However, the molecular mechanisms underlying the effect of hypoxia-induced reprogramming of iron metabolism on chemosensitivity in CRC remain unclear.

**Methods:**

RIP-seq combined with lncRNA-seq was conducted on hypoxia or normoxia CRC cells, to screen a profile of lncRNAs. Based on the characteristics of IRE elements, the sequence alignment and secondary structure analysis of the above screened lncRNAs were carried out by using open-access databases. Then lnc-IRP was obtained. RNA pull-down, RIP, RNA FISH and IF were used to confirm the binding and co-localization relationship between lnc-IRP and IRP2. Subsequently, the role of lnc-IRP in the chemosensitivity of CRC was verified both in vitro and in vivo.

**Results:**

lnc-IRP relied on its IRE-like element to competitively bind IRP2, which disrupted intracellular iron regulatory network, leading to a tendency of iron to be stored in a ferric form rather than converted to LIP, and ultimately inhibiting apoptosis and ferroptosis.

**Conclusions:**

lnc-IRP is a potential therapeutic target to restore iron homeostasis under hypoxia, thereby increasing the sensitivity of CRC to oxaliplatin, which is expected to provide a new choice for clinical treatment, and thus improve the prognosis of patients.

**Supplementary Information:**

The online version contains supplementary material available at https://doi.org/10.1186/s12967-026-08440-3.

## Introduction

According to the GLOBOCAN 2022 estimates of cancer incidence and mortality, colorectal cancer (CRC) is the third most commonly diagnosed cancer and the second leading cause of cancer-related death [1]. About one fifth CRC cases is diagnosed at an advanced stage, and the 5-year survival for these metastatic CRC (mCRC) patients is less than 15% [2]. Cytotoxic chemotherapy is the backbone of mCRC treatment, and currently available chemotherapeutic drugs remain limited [3]. The process of developing new drugs is complicated and time-consuming, so finding strategies to increase the efficacy of existing drugs remains critical.

Oxaliplatin, a classic chemotherapy agent for mCRC patients, interferes with replication and transcription via the formation of toxic covalent platinum-DNA adducts, ultimately leading to DNA damage. DNA repair and damage-bypass mechanisms are triggered immediately. Once DNA damage is not properly repaired, cells are finally pushed into death [4]. In addition, it has been shown that elevated reactive oxygen species (ROS) levels can increase the sensitivity to oxaliplatin [5, 6]. The dose-limiting peripheral neurotoxicity is a prominent characteristic of oxaliplatin. And patients often discontinue treatment because they cannot tolerate neurological dysfunction [7–9]. Therefore, it is not feasible to increase the efficacy of oxaliplatin by simply increasing the dosage.

Gastrointestinal bleeding is a common clinical symptom of CRC, resulting in anemia and iron deficiency, and causing the imbalance of iron homeostasis [2, 10, 11]. Research shows that about 60% of CRC patients have concomitant iron deficiency [11]. Iron deficiency promotes cancer progression by weakening immune surveillance or reshaping the immune microenvironment. However, the effect of iron deficiency on the intracellular biological function of CRC cells remains unclear.

Ferrous and ferric states are the main forms of biological iron. Ferric iron is mainly stored in ferritin. While free ferrous iron constitutes the cytosolic free labile iron pool (LIP), which has strong redox activity and can induce the generation of numerous ROS, thus promoting cell death processes such as necrosis, apoptosis and ferroptosis [12–16]. As the basal ROS content in cancer cells is higher than that in normal cells, further up-regulation of ROS to toxic levels is a key strategy for anticancer therapy [17]. Therefore, we hypothesized that increasing LIP accumulation to promote ROS production could serve as a potential strategy to enhance the cytotoxicity of oxaliplatin against CRC.

The maintenance of iron homeostasis at the whole-body level depends on hepcidin which is synthesized and secreted by the liver [18, 19]. Cellular iron metabolism is regulated by the iron regulatory protein / iron response element (IRP/IRE) system [20, 21]. IRE is a highly conserved stem-loop structure that is composed of 25–30 nucleotides in the untranslated region (UTR) of mRNA related to iron metabolism. The binding of IRP to IRE elements in the 5 ‘UTR of the target mRNA results in translation inhibition. Conversely, the binding of IRP to IRE elements in the 3 ‘UTR of the target mRNA brings about translational enhancement. Ultimately, IRP increases iron uptake and utilization of iron and decreases iron storage by binding to IRE in the 3’ or 5’ UTR region of downstream target mRNAs [22]. Compared with bifunctional IRP1, IRP2 is structurally unbound to iron-sulfur clusters and is more stable at the protein level [23]. In addition, IRP1/IRP2 knockout mice confirmed the dominance of IRP2 in iron homeostasis [24, 25]. Thus, our study mainly focused on IRP2.

The rapid proliferation of cancer cells combined with the abnormal structure and function of tumor blood vessels leads to inadequate oxygen supply within solid tumors [26, 27]. A series of adaptations associated with hypoxia occur in cancer cells, including the reprogramming of multiple metabolic pathways. Hypoxia is a major cause of poor therapeutic response of solid tumors. The extracellular matrix is remodeled within the hypoxic microenvironment, making drug delivery more difficult [28]. Moreover HIF-1α regulates the expression of genes involved in drug efflux, apoptosis, among others, ultimately reducing the effectiveness of drugs [29]. However, the effect of hypoxia-induced changes in iron metabolism on the chemosensitivity of CRC and the underlying mechanisms need to be further investigated.

With the development of high-throughput sequencing technology, the functions of non-coding RNAs (ncRNAs) have been gradually explored, among which miRNAs, circRNAs and lncRNAs are the most widely studied [30, 31]. LncRNAs are linear RNAs with a length greater than 200 nucleotides, which are longer than miRNAs and are not constrained by the closed-loop structure of circRNAs, thus they possess more diversified secondary structures [32]. Moreover, IRE-like elements are more likely to be found on lncRNA.

The purpose of this study was to screen hypoxia-associated lncRNAs with IRE-like elements and explore the mechanism by which lnc-IRP regulates chemosensitivity through interfering with the IRP/IRE system by competitively binding to IRP2 via its IRE-like element. This study also aimed to investigate the possibility of targeting lnc-IRP as a novel strategy to reverse hypoxia-induced iron homeostasis imbalance, and to increase the sensitivity to oxaliplatin of CRC.

## Materials and methods

### Human tissue

All cancer tissues were obtained from patients with newly diagnosed advanced CRC after palliative surgery at the Tianjin Medical University Cancer Institute and Hospital (Tianjin, China). All specimens were pathologically confirmed to be adenocarcinoma. All patients were classified as stage IV according to the TNM system designed jointly by the UICC (Union for International Cancer Control) and the AJCC (American Joint Committee on Cancer), and received oxaliplatin-based first-line chemotherapy. The informed consent was obtained from all participants. Ethical approval was granted by the Ethics Committee of Tianjin Medical University Cancer Institute and Hospital.

### Animals

Female nude mice (BALB/c-nu, 4 weeks) were purchased from the Beijing Vital River Laboratory Animal Technology Co., Ltd. Animals fed in an SPF (specific pathogen-free) animal facility. All of the experimental procedures were performed in accordance with protocols approved by the Institutional Animal Care and Research Advisory Committee of Tianjin Medical University Cancer Institute and Hospital.

### Cell culture

Human colorectal cancer cell lines SW480, HCT116, HT-29, Caco-2 were purchased from the Chinese Academy of Sciences (Shanghai, China), and LS180 was purchased from National Biomedical Cell-Line Resource Center (Beijing, China). SW480, HCT116, HT-29 and LS180 were cultured in 1640 (Gibco, USA) with 10% fetal bovine serum (FBS, Gibco, USA) and 1% penicillin/streptomycin. Caco-2 were cultured in DMEM medium (Gibco, USA) with 20% fetal bovine serum (FBS, Gibco, USA) and 1% penicillin/streptomycin. Cells were incubated in a humidified incubator at 37 °C with 5% CO_2_. Hypoxic culture refers to a reduction in oxygen concentration to 1%.

### Cell transfection

HCT116 and LS180 were seeded into 6-well plates and transfected using Lipofectamine 2000 (Invitrogen) and Opti-MEM (Gibco, USA) in accordance with the manufacturers’ instructions. ASO targeting lnc-IRP (RiboBio, Guangzhou, China; target sequence: 5’- ATGTCCCTTTTGCCAAAACCG − 3’) was applied to knock down lnc-IRP. Lnc-IRP and lnc-IRP Mut overexpression (OE) plasmids (GeneChem, Shanghai, China) were utilized to upregulate lnc-IRP / lnc-IRP Mut. Four to six hours after transfection, cells were washed twice with 1×PBS and subsequently replaced with complete medium. In addition, HCT116 and SW480 were harvested 24–48 h after transfection for downstream experiments, including RNA extraction, protein extraction, iron staining, Calcein-AM/PI double staining and so on.

### RNA isolation and quantitative RT-PCR

Total RNA was extracted from the cultured cells and tissues by TRIzol reagent (Invitrogen) according to the manufacturer’s protocols. A total of 1,000 ng of RNA was adopted to synthesize cDNA for reverse transcription PCR (Eppendorf AG 22331 Hamburg, Germany) to synthesize cDNA with avian myeloblastosis virus (AMV) reverse transcriptase (TaKaRa, Osaka, Japan). Then 1 µl of cDNA was used for real-time qPCR (Bio-Rad CFX96, Hercules, CA, USA). β-actin was utilized to normalize the level of lncRNA and mRNA. The Eq. 2^−ΔCt^ was used to calculate the relative quantities of target genes, ΔCt (cycle threshold) = Ct_gene_ -Ct_control_. All genes were assayed at least in triplicate.

The related primers were as follows:

Lnc-IRP / Lnc-IRP Mut primers

Forward 5’ - ACGCCTCCTACGTTTACCTG − 3’

Reverse 5’ - AGGCAAAAGCCCAGTGTATC − 3’

Lnc-SLC40A1-3:3 primers

Forward 5’ - CGCTTCCATAAGGCTTTGCC − 3’

Reverse 5’ - ATCCTCCTGGAACAACCACG − 3’

lnc-IFFO1-3:1 primers

Forward 5’ - ATGTGGGCCATGAGCTTGAC − 3’

Reverse 5’ - TGACTTCAACAGCGACACCC − 3’

β-actin primers

Forward 5’ - GGCTGTGCTATCCCTGTACG − 3’

Reverse 5’ - CTTGATCTTCATTGTGCTGGGTG − 3’

### Protein extraction and western blotting (WB)

SDS lysis buffer were prepared for protein extraction. Subsequently, the lysates were heated for 10 min at 95 °C to denature, cooled down for 5 min at room temperature (RT), and quantified by a NanoDrop 2000 (Thermo, Waltham, MA, USA). After loaded with β-Blue, 50 micrograms of lysates were separated on SDS-PAGE gels and transferred onto PVDF membranes (0.22 μm; Roche, Basel, Switzerland). The membrane was cut according to the position of markers and the molecular weight of target protein. And the bands were blocked with 5% BSA at RT for 1 h and incubated at 4 °C overnight with anti-HIF-1 (1:1000, Abcam), anti-IRP2 (1:500, Santa Cruz), anti-TFRC (1:3000, Abcam), anti-Ferritin (1:2000, Abcam), anti-PTGS2 (1:500, Santa Cruz), anti-FTH1 (1:500, Santa Cruz), anti-Caspase3/cle- Caspase3 (1:5000, Abcam), anti-BAX (1:500, Santa Cruz), anti-BCL2 (1:500, Santa Cruz), anti-β-actin (1:3000, Santa Cruz), respectively. The expression of protein was normalized to β-actin. After incubation with the secondary antibody (Abcam), the bands were visualized with an Enhanced Chemiluminescence System Kit (Millipore, USA).

### RNA-Seq and RIP-Seq

HCT116 cells were seeded into 6 cm dishes, and cultured under normoxic or hypoxic conditions for 48 h. And then, after washed twice with 1× PBS, cells were fully lysed with TRIzol reagent and frozen in liquid nitrogen for lncRNA-seq.

HCT116 cells were seeded into 10 cm dishes, and cultured under normoxic or hypoxic conditions for 48 h. Then cells were washed twice and scraped into 2 mL of prechilled 1×PBS. Subsequently, the collected mixture was centrifuged at 1000 rpm for 5 min at 4°C. The precipitate was resuspended by prechilled 1×PBS, then centrifuged again at 1000 rpm for 5 min at 4°C, and repeated once. After discarding the supernatant, 150 µL of lysate (5 mM MgCl_2_, 10 mM HEPES-NaOH pH 7, 100 mM KCl, 0.5% NP-40, 200 U/mL RNase OUT, Cocktail, 1 mM DTT) was added to the precipitate, lysed on ice for 5 min, and stored at -80°C for further lysis. The 75 µL of protein A/G magnetic beads (Thermo, Massachusetts, USA) were washed with 1×NT-2 buffer (150 mM NaCl, 50 mM Tris-HCL pH 7.4, 1 mM MgCl_2_, 0.05% NP-40) and then incubated with 5 µg of IRP2 antibody (Santa Cruz) for 1 h at RT. Then the above mixture was centrifuged at 5000 g for 15 s at RT. After collection on a magnet, the incubated beads were washed five times with 1×NT-2 buffer, subsequently resuspended in 900 µL of NET-2 buffer (addition of 200 U/mL RNase OUT, 1 mM DTT and 20 mM EDTA pH 8.0 into 1×NT-2) and placed on ice. The lysates above that stored at -80°C were melted on ice and then centrifuged at 20,000 g for 10 min at 4°C. And 10 µL supernatant was stored as ‘input’ at -80 °C. Another 100 µL of the supernatant was added to the magnetic beads coated with IRP2 antibody (900 µL, 1 mL in total) and mixed overnight at 4 °C. Subsequently, the magnetic beads were collected by placing the mixture on a magnet for 1 min, washed six times with 1×NT-2 buffer oscillatory. Finally, the RIP sample was collected. RNA was extracted by phenol-chloroform method from the RIP sample and frozen in liquid nitrogen for RIP-seq.

All of the sequencing and bioinformatics analyses were performed by Gene Denovo Biotechnology Co., Ltd. (Guangzhou, China).

### RNA Pull-down assay

HCT116 cells cultured in 10 cm dishes were washed twice with prechilled 1×PBS, and scraped into cell lysis buffer. The lysate was repeatedly freeze-thawed in liquid nitrogen five times, then centrifuged at 3000 rpm for 10 min at 4 °C. The supernatant was collected carefully, mixed with 200 U/mL RNase inhibitor and stored at -80℃. And 10 µL of lysates was stored as ‘input’.

The DynabeadsTM MyOneTM Streptavidin C1 (Invitrogen, USA) were washed and mixed with 400 µL 2×B&W buffer, 50 pmol biotin-modified RNA (lnc-IRP-biotin or control-biotin) that synthesized by RiboBio (Guangzhou, China) and 280 µL water-DEPC treated, rotated for 20 min at RT, then further incubation for 30 min at RT. Magnetic beads attached to RNA-biotin were collected with the help of a magnet, and washed three times with 1×B&W. The 500 µL of cell lysates above and 1 U/µL RNase inhibitor were added into these prepared magnetic beads, rotated overnight at 4℃. And then the magnetic bead complex was collected. Proteins in the beads complex and ‘input’ were extracted for WB detection.

### FISH and immunofluorescence

HCT116 and LS180 cells were seed into 24-well plate and grown on glass coverslips. Cells were fixed with 4% PFA for 10 min, incubated in permeabilization buffer with Triton X-100 for 5 min at 4℃, and blocked in preheated (37℃) prehybridization solution, then hybridized with lnc-IRP FISH Probe Mix solution (RiboBio, Guangzhou, China) overnight in the dark at 37℃. The following steps were performed without light. The hybridized cells were washed sequentially with 4×SCC, 2×SCC, 1×SCC buffer. Subsequently, the cells were blocked in 2% BSA solution for 1 h at RT, incubated with IRP2 antibody overnight at 4℃, incubated with Alexa 488-conjugated secondary antibody for 1 h at RT, and stained with DAPI for 10 min at RT. After sealing with antifade mounting medium, the slices were observed under a laser confocal microscope.

### Determination of iron content

#### Total iron

Total iron of CRC cells and tissue was detected with the Total Intracellular Iron Assay Kit and Tissue Iron Content Assay Kit (Solarbio, Beijing, China), according to the manufacturer’s instructions. Firstly, Fe^3+^ was reduced to Fe^2+^ by sodium sulfite. And Fe^2+^ further reacts with 2,2-bipyridine to create an orange complex which has an absorption peak at 520 nm. The total iron content can be calculated by measuring the absorbance at 520 nm.

#### Ferrous iron

Ferrous iron of CRC cells was quantitative detected with the Ferrous iron Assay Kit in accordance with the manufacturer’s instructions (Solarbio, Beijing, China).

The content of Fe^2+^ in alive cells was measured by FerroOrange probe (Dojindo, Japan). CRC cells were seed into 24-well plate and grown on glass coverslips. After wash with 1× PBS, cells were incubated with FerroOrange probe for 45 min at 37℃. After sealing with antifade mounting medium, the slices were observed under a laser confocal microscope. The content of Fe^2+^ in CRC tissue was tested by Turnbull’s blue reaction. The kit purchased from Beijing Solarbio Science & Technology Co., Ltd. The experimental procedure was according to the instructions.

The staining reaction is as follows: 3Fe^2+^+2[Fe(CN)_6_]^3−^= Fe_4_[Fe(CN)_6_]_3_↓.

#### Ferric iron

The level of Fe^3+^ in cells and tissues was analyzed by Perls’ staining (Kit from Solarbio, Beijing, China) according to the manufacturer’s instructions.

The staining reaction is as follows: 4Fe^3+^+3[Fe(CN)_6_]^4−^= Fe_4_[Fe(CN)_6_]_3_↓.

### Calcein-AM / PI double staining

Cells were seed into 24-well plate and grown on glass coverslips. At harvest, both the attached and floating cells were collected and stained. Calcein-AM can penetrate the cell membrane and enter into living cells. then its AM group is removed by esterase to become Calcein with strong green fluorescence. PI (propidium iodide) can be embedded in the DNA double helix of dead cells, showing red fluorescence. Therefore, calcein-AM / PI double staining can be used to analyze the status of cell survival or death (Kit from Dojindo, Japan). After sealing, the slices were observed under a fluorescent microscope.

### CCK-8 cell viability assay

After counting, cells were seeded in 96-well plates at a density of 5000 to 8000 cells per well and followed by different pretreatments. Cell viability was detected with CCK-8 (Solarbio). The optical density (OD) value was examined by a microplate reader (Thermo) at a wavelength of 450 nm.

### Cell apoptosis assay

Cells were cultured in 6-well plates followed by different pretreatments. Subsequently, both the attached and floating cells were collected and stained with Annexin V-FITC and PI (BD Biosciences, Franklin Lakes, NJ, USA). The result was quantified by a fluorescence-activated cell sorting flow cytometer (BD Biosciences).

### MDA analysis

Cells were cultured in 6-well plates followed by different pretreatments. Intracellular MDA (malondialdehyde) were isolated and tested by MDA Content Assay Kit according to the manufacturer’s protocol (Solarbio, Beijing, China). The MDA levels were calibrated with the number of cells.

### ROS and LPO measurement

#### ROS detection

Cells were cultured in 6-well plates followed by different pretreatments. After washed with 1×PBS, cells were incubated with 10 µM 2,7-dichlorofluorescein diacetate (DCFH-DA, Solarbio) for 30 min, then digested with trypsin and resuspended, and analyzed at a wavelength of 488 nm by a fluorescence-activated cell sorting flow cytometer (BD Biosciences).

#### LPO (Lipid-ROS) detection

Cells were cultured in 6-well plates followed by different pretreatments. After washed with 1×PBS, cells were incubated with 5 µM BODIPY TM 581/591 C11 Lipid Peroxidation Sensor (Invitrogen) for 30 min, then digested with trypsin and resuspended, and analyzed at excitation wavelengths of 581 nm and 500 nm by a fluorescence-activated cell sorting flow cytometer (BD Biosciences).

### Establishment of tumor xenografts in mice

Lentivirus were bought from Shanghai Genechem Co. Ltd. And used to infect HCT116 cells and obtain stable higher expression of lnc-IRP, lnc-IRP Mut, and lower expression of lnc-IRP and control HCT116 cells. Then, those cells were inoculated subcutaneously into in the inguinal regions of nude mice (0.8 × 10^7^ cells, 6 mice per group). From the sixth day of tumor implantation, the symptoms of gastrointestinal blood loss in CRC patients were simulated according to the establishment method of hemorrhagic anemia model, that is, a small amount of bloodletting from the tail vein of mice for multiple times, each time 0.01 mL/g, once every 6 days until the experiment was completed. At the same time, oxaliplatin 5 mg/kg was injected intraperitoneally every 4 days from the eighth day of tumor implantation. Except for the model control group (oxaliplatin treatment group), the other four groups of mice were given oxaliplatin treatment and supplemented with FeSO_4_ at a dose of 50 mg/kg/day. ELISA assay was used to measure the hemoglobin concentration of mice (Shanghai Enzyme-linked Biotechnology Co., Ltd).

### Statistical analysis

The quantification for staining-based assays (such as iron staining and fluorescence imaging) is performed using ImageJ and conducted in a blinded manner. All the data in our study are based on at least three independent experiments and are presented as the mean ± SD. The t-test was used to analyze the significance of the differences between groups. Differences were considered statistically significant at *P* < 0.05. In this study, ‘*’ indicates *P* < 0.05, ‘**’ indicates *P* < 0.01.

## Results

### Oxygen availability regulates iron‑mediated oxaliplatin sensitization and the IRP/IRE system function

To a certain extent, the cytotoxic effect of oxaliplatin is regulated by ROS levels [5, 6]. LIP triggers massive ROS generation via the Fenton reaction. Therefore, this study explored the effect of iron supplementation on oxaliplatin sensitivity in CRC and whether this effect is modulated by oxygen supply intensity.

**Fig. 1:**
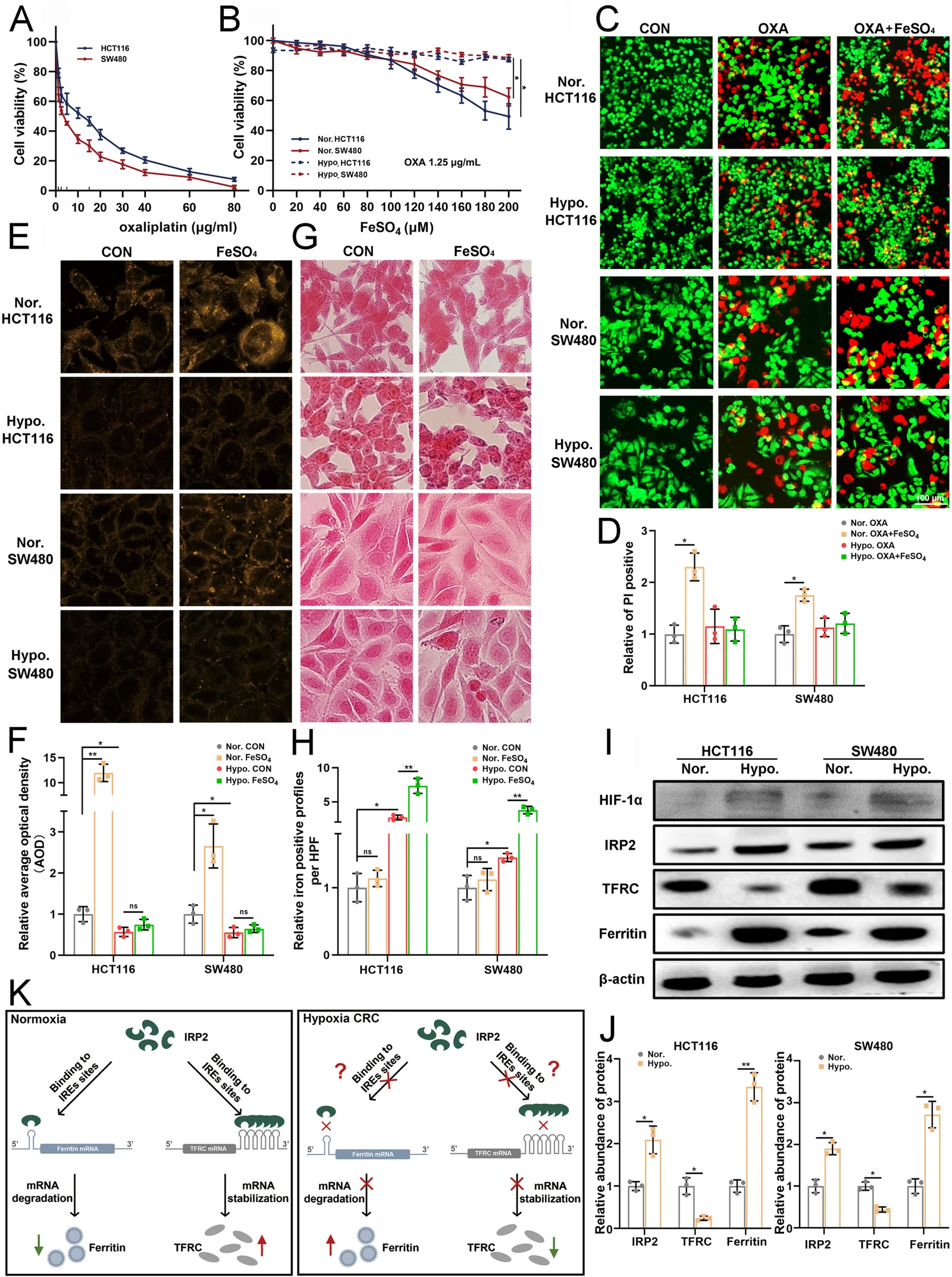
Iron supplementation increased the sensitivity of CRC cells to oxaliplatin, but failed in hypoxic environments. (**A**) Cell viability detection of HCT116 and SW480 that treated with OXA. (**B**) Iron supplementation on OXA sensitivity of HCT116 and SW480 under normoxic or hypoxic condition. (OXA 1.25 µg/mL) (**C**) Verification of the effect of iron supplementation on cell survival in normoxic or hypoxic culture via Calcein-AM/PI double stain. Living cells were labeled green by Calcein-AM, and dead cells were labeled red by PI. (OXA 1.25 µg/mL, FeSO4 180 µM) (**D**) Quantitative analysis of (**C**). (**E**) FerroOrange probe was used to detect the intracellular content of LIP after the addition of FeSO4. (FeSO4 180 µM) (**F**) Quantitative analysis of (**E**). (**G**) Perls’ staining was used to test the intracellular content of ferric iron after the addition of FeSO4. (FeSO4 180 µM) (**H**) Quantitative analysis of (**G**). (**I**) The expression of IRP2, TFRC and Ferritin in CRC cells was measured by WB. (**J**) Quantitative analysis of (**I**). (**K**) Briefly graphical representation of IRP/IRE regulatory system in CRC cells under normoxic or hypoxic condition. (‘Nor’ indicates ‘Normoxia’, ‘Hypo’ indicates ‘Hypoxia’, ‘CON’ indicates ‘Control’, ‘OXA’ indicates ‘Oxaliplatin’, ‘LIP’ indicates ‘Labile iron pool’, ‘ns’ indicates ‘no statistical differences’, ‘*’ indicates ‘P < 0.05’, ‘**’ indicates ‘P < 0.01’, Data are presented as mean ± SD, “N” ≥ 3)

The sensitivity of two CRC cell lines to oxaliplatin was assessed using CCK-8 assay (Fig. 1A). An oxaliplatin concentration of 1.25 µg/mL was chosen for subsequent experiments to ensure that the vast majority of HCT116 and SW480 cells (over 60%) remained viable. As shown in Fig. 1B, iron supplementation enhanced oxaliplatin sensitivity in HCT116 and SW480 cells under normoxic conditions. However, this sensitizing effect was attenuated or even abolished under hypoxic conditions. A concentration of 180 µM FeSO_4_ was selected for subsequent experiments owing to its marked chemosensitizing effects and favorable stability in cell culture medium. Calcein-AM / PI double staining further verified that iron supplementation elevated the proportion of PI-positive cells under normoxia, whereas no such change was observed under hypoxia (Fig. 1C and D). These findings suggest that iron supplementation may fail to increase LIP levels in CRC cells under hypoxic conditions.

The FerroOrange probe was employed to detect the content of ferrous iron or LIP in live cells. As shown in the Fig. 1E and F, LIP content in CRC cells cultured under hypoxia was decreased, and it was barely increased following iron supplementation. The results of Perls’ staining revealed that the content of ferric iron in CRC cells under hypoxia was higher than that under normoxia, and it was further increased after iron supplementation (Fig. 1G and H).

IRP / IRE, which maintains intracellular iron homeostasis, was first described in the 1980s [33]. IRE elements were first identified in the UTR region of the mRNAs encoding transferrin receptor (TFRC) and ferritin. TFRC is necessary for cellular iron uptake. In contrast, ferritin is a protein that stores iron in a ferric form. The results of WB showed that the expression of IRP2 was increased in HCT116 and SW480 cells under hypoxic conditions, while the expression of TFRC was decreased and ferritin was up-regulated (Fig. 1I and J).

Under normal conditions, the binding of IRP2 to IRE in the 5’UTR region of ferritin mRNA suppresses its expression, while the binding of IRP2 to IRE in the 3’UTR region of TFRC mRNA enhances TFRC expression. However, under hypoxic conditions, the expression of IRP2 was augmented, but the level of TFRC was not elevated and ferritin levels were not reduced. These results suggest that the post-transcriptional regulatory function of IRP2 is limited in hypoxic CRC cells, and the increased IRP2 appears unable to bind to the IRE element of its downstream targets. We hypothesized that iron metabolism in hypoxic CRC cells might be regulated by a lncRNA containing IRE-like elements, which competitively binds to IRP2 and impairs its function (Fig. 1K).

### High-throughput screening of lncRNA containing IRE-like elements and associated with hypoxia

**Fig. 2:**
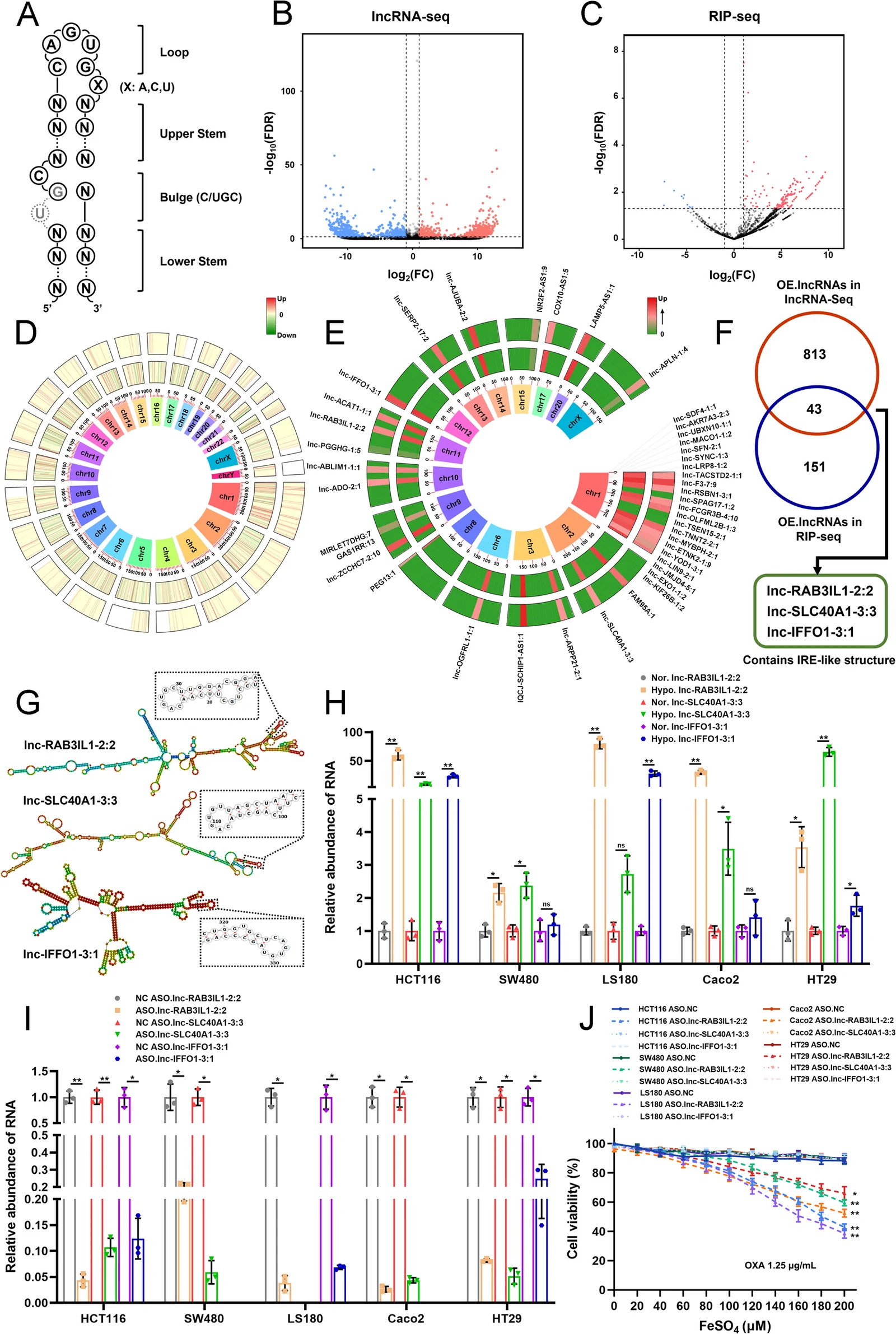
High-throughput sequencing of lncRNAs associated with hypoxia and containing IRE-like elements. (**A**) Schematic diagram of the IRE element structure. (**B**) Volcano plot of differentially expressed lncRNAs between Nor.HCT116 and Hypo.HCT116 by lncRNA-seq. (**C**) Volcano plot of differentially expressed lncRNAs between groups by IRP2-binding RIP-seq. (**D**) Circos plot of differentially expressed lncRNAs in lncRNA-seq and IRP2-binding RIP-seq. Inner track: chromosomes (chr1–22, X, Y). Middle track: significantly dysregulated lncRNAs in hypoxia by lncRNA-seq. Outer track: Significantly dysregulated lncRNAs during hypoxia in IRP2-binding RIP-seq. (red: upregulated, green: downregulated). (**E**) Circos plot illustrating the intersection of lncRNA-seq and IRP2-binding RIP-seq overexpression profiles identifying 43 hypoxia-induced lncRNAs. Inner track: chromosomes (chr1–3, 6, 8–15, 17,20, X). Middle track: 43 lncRNAs in lncRNA-seq. Outer track: 43 lncRNAs in RIP-seq. (gradient represents upregulation level. greener: low, redder: high). (**F**) Three lncRNAs, lnc-RAB3IL1-2:2, lnc-SLC40A1-3:3 and lnc-IFFO1-3:1, were finally selected. (**G**) The secondary structure of lnc-RAB3IL1-2:2, lnc-SLC40A1-3:3 and lnc-IFFO1-3:1. (**H**) PCR verification of the 3 screened lncRNA in CRC cells under normoxia or hypoxia condition, among which the level of lnc-RAB3IL1-2:2, subsequently referred to as lnc-IRP, was the most stably upregulated in all hypoxic CRC cells. (**I**) PCR analysis of the 3 screened lncRNA in hypoxic CRC cells. (**J**) Cell viability detection of CRC cells upon knockdown of the three lncRNAs. (OXA 1.25 µg/mL). (‘Nor’ indicates ‘Normoxia’, ‘Hypo’ indicates ‘Hypoxia’, ‘OE’ indicates ‘Overexpression’, ‘ns’ indicates ‘no statistical differences’,‘*’ indicates ‘P < 0.05’, ‘**’ indicates ‘P < 0.01’, Data are presented as mean ± SD, “N” ≥3)

The IRE is a highly conserved hairpin structure of 25–30 nucleotides, which contains a typical loop with the sequence of CAGUGX (X denotes A, C or U) and a base-paired stem that is interrupted by an unpaired C residue or an asymmetric UGC/C bulge/loop (Fig. 2A). Normoxic and hypoxic HCT116 cells were utilized for lncRNA-seq and RIP-seq (Fig. 2B and C). Differential expression levels of lncRNAs between groups were analyzed with the criteria of ∣log2(FC)∣ ≥ 1 and FDR < 0.05 (Fig. 2D). By analyzing the sequencing results, a total of 43 lncRNAs that were higher expressed under hypoxic conditions and could bind to IRP2 were obtained (Fig. 2E).

Next, according to the characteristics of IRE elements, the primary structures of the above 43 lncRNAs were manually compared, and the sites containing 5’-CAGUGX-3’ were retrieved (Fig. 2F). The secondary structure was then checked using the online tool RNAfold WebServer (http://rna.tbi.univie.ac.at//cgi-bin/RNAWebSuite /RNAfold.cgi). Finally, three lncRNAs containing IRE-like elements were screened, including lnc-RAB3IL1-2:2, lnc-SLC40A1-3:3, and lnc-IFFO1-3:1 (Fig. 2G).

RT-qPCR was used to detect the expression of these three lncRNAs in hypoxic or normoxic CRC cells including SW480, HCT116, HT-29, Caco2 and LS180. As shown in Fig. 2H, compared with normal oxygen culture, the expression of lnc-SLC40A1-3:3 in hypoxic LS180 seemed to have an upward trend, but there was no statistical significance, and the expression of lnc-IFFO1-3:1 did not increase in hypoxic SW480 and Caco2. However, only lnc-RAB3IL1-2:2 was more stably up-regulated in all the above hypoxic CRC cells. Therefore, in the subsequent ASO‑mediated knockdown experiments under hypoxia, only the hypoxia‑upregulated lncRNAs in each cell line were knocked down, excluding the above‑mentioned non‑hypoxia‑induced lncRNA‑cell pairs.

ASO-mediated knockdown of lnc-RAB3IL1-2:2, lnc-SLC40A1-3:3, and lnc-IFFO1-3:1 was performed in hypoxic SW480, HCT116, HT-29, Caco2, and LS180, respectively. And the silencing efficiency was then validated by RT-qPCR (Fig. 2I). Subsequently, the sensitivity to oxaliplatin was evaluated by CCK-8 assay in hypoxic CRC cells following knockdown of the three aforementioned lncRNAs. Only upon downregulation of lnc-RAB3IL1-2:2 was a significant reduction in cell viability observed, indicating enhanced oxaliplatin sensitivity in the hypoxic CRC cells (Fig. 2J). It contains an IRE-like element and can bind to IRP2, and is referred to as lnc-IRP in this study. In addition, RT-qPCR results suggested that lnc-IRP was more significantly upregulated in LS180 and HCT116 under hypoxic culture. Therefore, subsequent in vitro studies will be carried out in these two CRC cell lines. SiRNA‑mediated knockdown of HIF expression was performed in hypoxic HCT116 and LS180 cells, followed by qRT‑PCR‑mediated quantification of lncRNA levels. The results showed that HIF‑1α depletion did not significantly attenuate hypoxia‑induced upregulation of lnc‑IRP. These findings indicate that the hypoxic induction of lnc‑IRP is largely independent of HIF‑1α (Fig. S1A).

### Validation that lnc-IRP reduces oxaliplatin sensitivity of CRC through iron metabolism in clinic

**Fig. 3:**
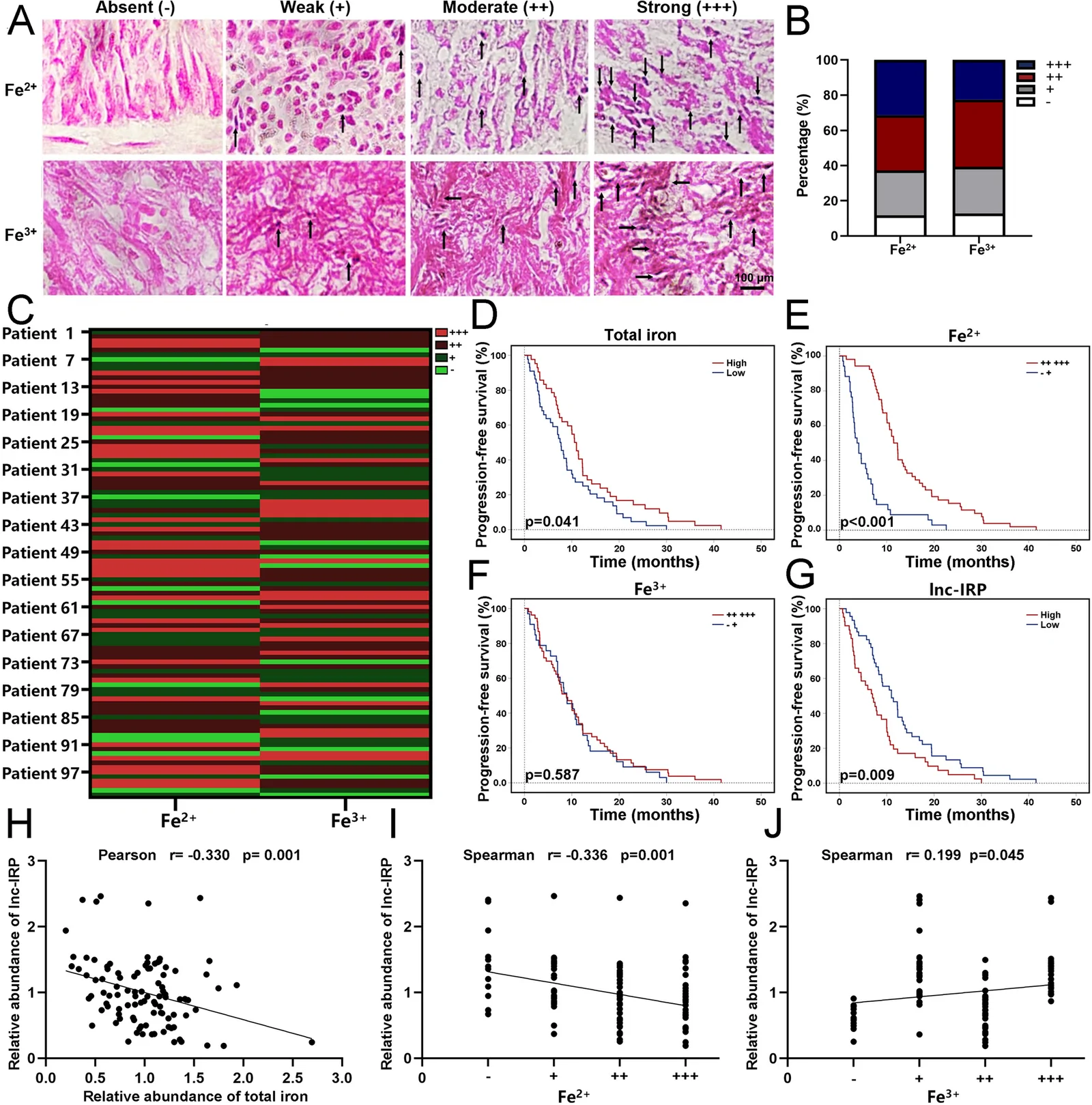
Clinical validation that lnc-IRP regulates oxaliplatin sensitivity of CRC through iron metabolism. (**A**) Turnbull’s blue reaction and Perls’ blue reaction were performed to detect ferrous iron and ferric iron in CRC tissues, respectively. Scale bar: 100 μm. Arrows indicate typical iron‑positive granules. Staining grades were classified according to standard pathological semiquantitative criteria (**B**) Percent stacked column showing the proportion of different iron‑staining grades in clinical CRC samples. (**C**) Heatmap displaying iron‑staining intensity distribution across patients. (**D**) The progression-free-survival curve for the level of total iron in CRC. (**E**) The progression-free-survival curve for the level of ferrous iron in CRC. (**F**) The progression-free-survival curve for the level of ferric iron in CRC. (**G**) The progression-free-survival curve for the level of lnc-IRP in CRC. (**H**) Pearson correlation analysis between lnc-IRP levels and the content of total iron. (**I**) Spearman correlation analysis between lnc-IRP levels and the content of ferrous iron. (**J**) Spearman correlation analysis between lnc-IRP levels and the content of ferric iron

In this study, palliative surgery specimens were collected from 102 mCRC patients who received oxaliplatin-based first-line chemotherapy. There were 55 males (53.9%) and 47 females (46.1%), ranging in age from 30 to 75 years old, with 48 cases under 60 years old (47.1%), and 54 cases over 60 years old (52.9%). There were 88 cases (86.3%) in the colon, 11 cases (10.8%) in the rectum, and 3 cases (2.9%) in the rectosigmoid junctions. The ferrous iron content was tested by Turnbull’s blue reaction and the ferric iron content was examined by Perls’ blue reaction. According to the proportion of positive particles (blue) and the staining intensity, the results were divided into four grades: absent, weak, moderate, and strong (Fig. 3A and C). Lnc-IRP in tissues was detected by RT-qPCR. And the total iron content was measured by bipyridine colorimetry (Table S1). Kaplan-Meier methods were used to analyze the effects of lnc-IRP, total iron, ferrous iron and ferric iron on the progression-free survival (PFS) of first-line treatment. As indicated in Fig. 3D, the median PFS (mPFS) of patients with higher total iron content was 10.57 months, while that of patients with lower total iron content was 7.53 months, with a statistically significant difference (*P* < 0.05). The mPFS of patients with a higher ferrous iron content is longer (11.43 months vs. 3.70 months, *P* < 0.05) (Fig. 3E). There was no statistically significant difference in mPFS between patients with high and low ferric iron content (8.87 months vs. 8.93 months, *P* > 0.05) (Fig. 3F). And overexpression of lnc-IRP shortened mPFS (7.00 months vs. 11.00 months, *P* < 0.05) (Fig. 3G). As shown in Fig. 3H–J, the expression of lnc-IRP was negatively correlated with total iron content (pearson correlation coefficient *r*= -0.330, *P* < 0.05) and ferrous iron content (spearman correlation coefficient *r*= -0.336, *P* < 0.05), and was positively correlated with ferric iron content (spearman correlation coefficient *r* = 0.199, *P* < 0.05).

Furthermore, clinicopathological characteristics and the four measured indicators were incorporated into the univariate model. Variables with statistical significance were further enrolled into the multivariate Cox regression model. The results verified that lnc-IRP and ferrous iron indexes remained independent prognostic factors for PFS after adjustment for other clinical confounding variables. Kaplan-Meier survival analysis revealed that patients with high total iron levels presented significantly better PFS than those with low total iron levels. Considering the iron metabolic characteristics of tumor cells, the improved prognosis in the high total iron group might be a secondary phenomenon caused by synchronized ferrous iron elevation, rather than an independent prognostic effect of total iron itself. Notably, all patients enrolled in this study were diagnosed with stage IV CRC with consistent tumor stage baseline. Therefore, tumor stage was not included in the subsequent confounding factor correction. After eliminating the interference of clinical baseline covariates in the Cox regression model, total iron showed no independent predictive value for patient survival, whereas ferrous iron maintained stable prognostic significance. This further verified that the redistribution of iron from inert ferric iron to biologically active ferrous iron is the core functional mechanism affecting the clinical outcome of advanced colorectal cancer (Table S2).

### Identification of the interaction between lnc-IRP and IRP2

**Fig. 4:**
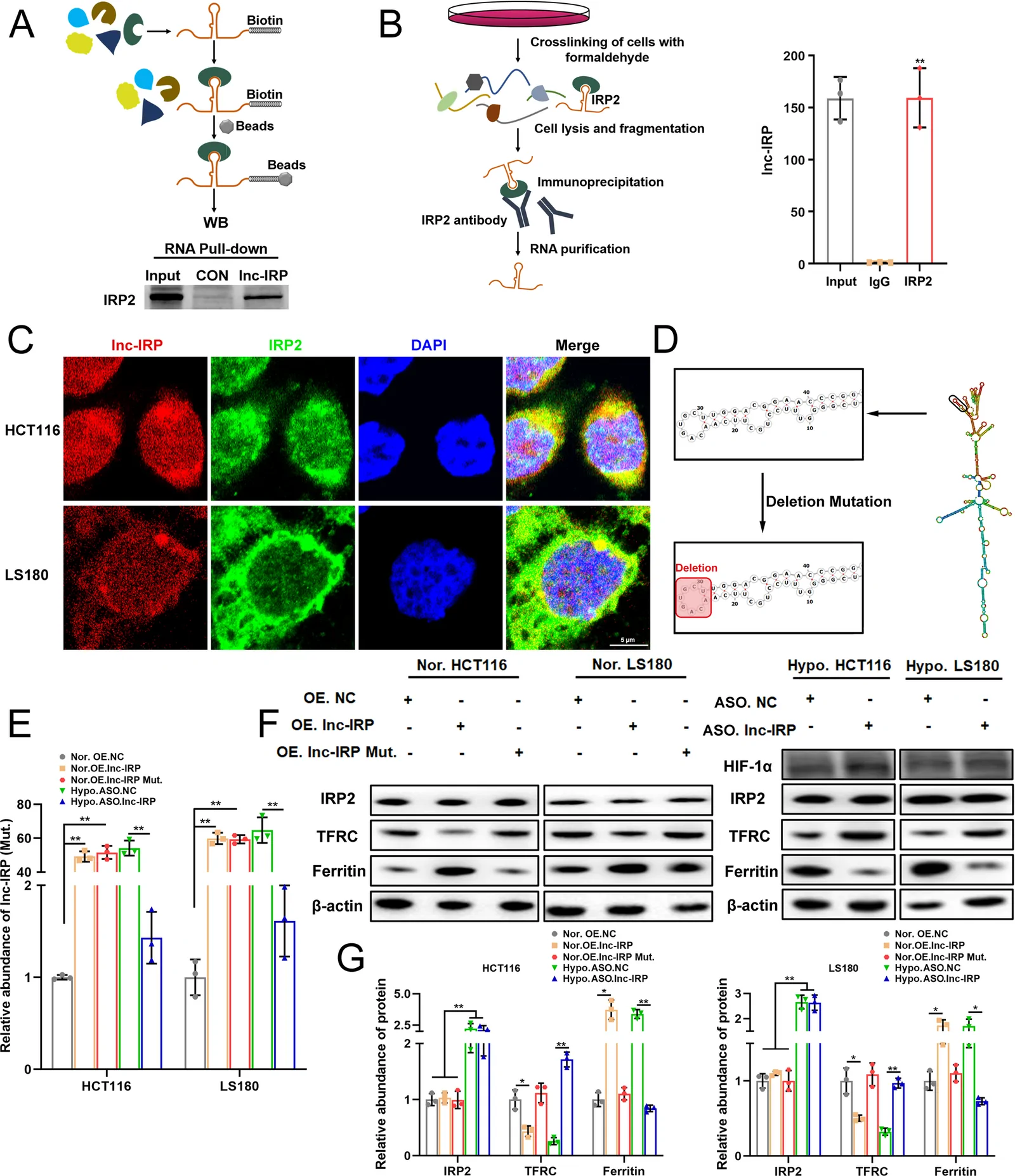
The effect of lnc-IRP on iron metabolism depends on IRE-like element. (**A**) RNA Pull-down assay confirmed the binding of lnc-IRP to IRP2. (**B**) RIP assay further confirmed that IRP2 binds to lnc-IRP. (**C**) RNA FISH and immunofluorescence demonstrated the co-localization of lnc-IRP and IRP2. (**D**) Schematic diagram of mutation of the IRE-like element and its location on the lnc-IRP. Specifically, lnc-IRP Mutation plasmid was constructed by deleting the characteristic circular sequence on the IRE-like element of lnc-IRP. (**E**) PCR analysis of the levels of lnc-IRP or lnc-IRP Mut in transfected HCT116 and LS180 cells under normoxic or hypoxic condition. (**F**) WB verification of the key iron-metabolism related proteins attenuation and restoration in each group, including IRP2, TFRC and Ferritin. (**G**) Quantitative analysis of (**F**). (‘Nor’ indicates ‘Normoxia’, ‘Hypo’ indicates ‘Hypoxia’, ‘CON’ indicates ‘control’, ‘OE’ indicates ‘Overexpression’, ‘ASO’ indicates ‘Antisense oligonucleotide’, ‘NC’ indicates ‘Negative control’, ‘Mut’ indicates ‘Mutation’, ‘*’ indicates ‘P < 0.05’, ‘**’ indicates ‘P < 0.01’, Data are presented as mean ± SD, “N” ≥3)

The biotin-labeled lnc-IRP was coincubated with the lysate of HCT116, and the results showed that IRP2 was enriched in the product of lnc-IRP-biotin pull-down, which preliminarily confirmed that lnc-IRP could bind with IRP2 (Fig. 4A). Subsequently, the IRP2-specific antibody and Protein-A/G-coated magnetic beads complex were coincubated with the cell lysate of HCT116, and the elution products were enriched with lnc-IRP, further confirming the interaction between IRP2 and lnc-IRP (Fig. 4B). RNA FISH and immunofluorescence verified the colocalization of lnc-IRP and IRP2 (Fig. 4C). RNA‑RIP‑qPCR assays demonstrated that lnc‑IRP competitively bound to IRP2 and displaced IRP2 from its canonical downstream target TFRC mRNA and ferritin mRNA (Fig. S1B). The plasmid that overexpresses lnc-IRP or its IRE-like element mutation (referred to as lnc-IRP Mut, Fig. 4D) was transfected into normoxic HCT116 and LS180, and ASO lnc-IRP was transfected into hypoxic HCT116 and LS180. The transfection efficiency was confirmed by RT-qPCR (Fig. 4E). The expressions of IRP2, TFRC and Ferritin were detected by WB. As shown in Fig. 4F and G, the expression of IRP2 remained basically constant in all groups under the same oxygen supply state (normoxia or hypoxia), but the contents of downstream proteins TFRC and Ferritin changed with the level of lnc-IRP but not its mutant.

### The effect of lnc-IRP on the sensitivity to oxaliplatin via the IRE-like element

**Fig. 5:**
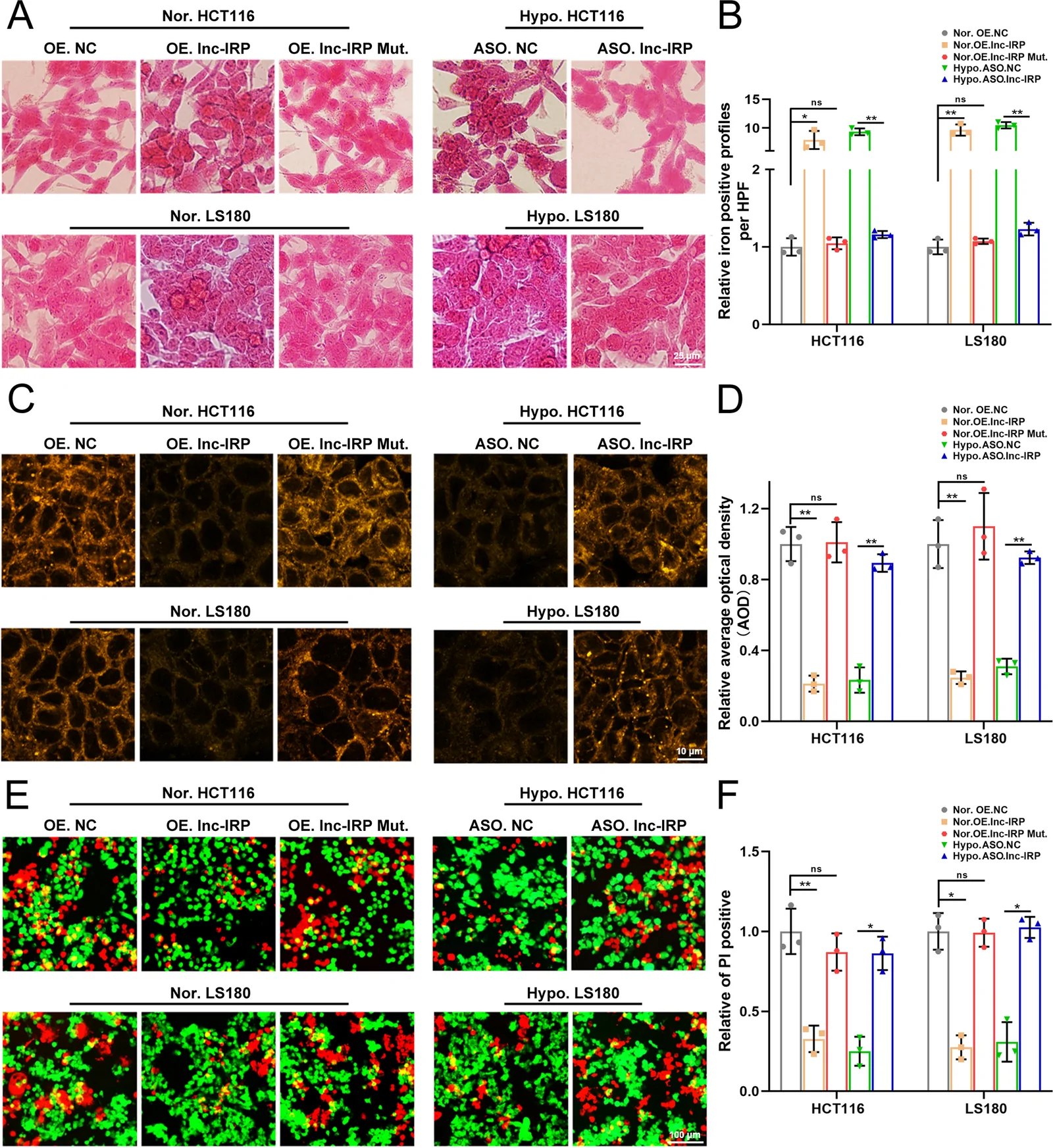
lnc-IRP regulates chemosensitivity to oxaliplatin of CRC by interfering with iron metabolism. (**A**) Perls’ blue reaction was used to measure ferric iron in HCT116 and LS180 cells. (**B**) Quantitative analysis of (**A**). (**C**) FerroOrange probe was applied to analyze the level of ferrous iron in HCT116 and LS180 cells. (**D**) Quantitative analysis of (**C**). (**E**) Calcein-AM/PI double staining was adopted to evaluate the survival or death status of HCT116 and LS180 cells in each group. (**F**) Quantitative analysis of (**E**). (‘Nor’ indicates ‘Normoxia’, ‘Hypo’ indicates ‘Hypoxia’, ‘OE’ indicates ‘Overexpression’, ‘ASO’ indicates ‘Antisense oligonucleotide’, ‘NC’ indicates ‘Negative control’, ‘Mut’ indicates ‘Mutation’, ‘ns’ indicates ‘no statistical differences’, ‘*’ indicates ‘P < 0.05’, ‘**’ indicates ‘P < 0.01’, Data are presented as mean ± SD, “N” ≥3)

HCT116 and LS180 were still divided into the above five groups and treated with iron (FeSO_4_ 180 µM). They were negative control for overexpression group (OE NC), lnc-IRP overexpression group (OE lnc-IRP), lnc-IRP Mut overexpression group (OE lnc-IRP Mut), negative control for downregulation group (ASO NC), lnc-IRP downregulation group (ASO lnc-IRP). The first three groups were cultured under normal oxygen conditions, and the last two groups were cultured under hypoxic conditions. FerroOrange probe and Perls’ blue staining method were used to detect the content of ferrous and ferric iron, respectively (Fig. 5A and D). In addition, visible spectrophotometry was utilized to quantify intracellular total iron, ferrous iron, and ferric iron, with ferric iron calculated as total iron minus ferrous iron (Fig. S1C-S1F). The results showed that upregulation of lnc-IRP containing complete IRE-like elements could increase the content of ferric iron and decrease the content of ferrous iron in normoxic CRC cells. At the same time, knocking down lnc-IRP could reverse the accumulation of ferric iron in hypoxic condition and augment the ferrous form.

In the above groups, oxaliplatin (1.25 µg/mL) was added at the same time of iron supplement. Subsequently, the survival / death status of the cells was analyzed by Calcein-AM / PI double staining. As shown in Fig. 5E and F, compared with the control group (OE NC), the proportion of PI-positive staining in the OE lnc-IRP group decreased, while there was no significant change in the OE lnc-IRP Mut group. On the contrary, the PI positive staining increased in ASO lnc-IRP group in comparison with control group (ASO NC) under hypoxic condition.

### The lnc-IRP / IRP2 pathway weakens oxaliplatin sensitivity by inhibiting apoptosis and ferroptosis

**Fig. 6:**
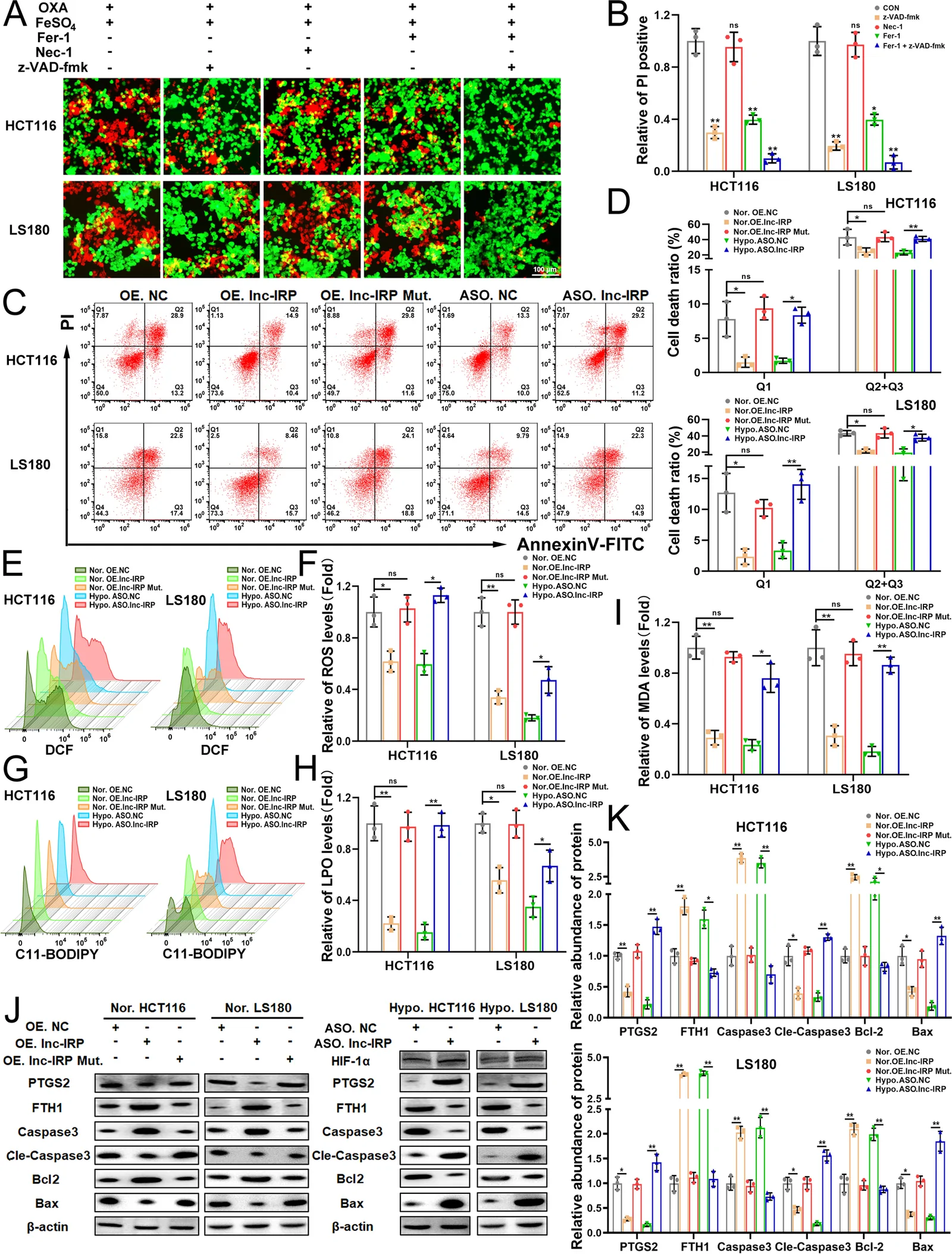
Lnc-IRP / IRP2 / iron-metabolism pathway attenuates oxaliplatin sensitivity of CRC by inhibiting apoptosis and ferroptosis. (**A**) Normoxic HCT116 and LS180 cells were supplemented with iron (180 µM) and added with ferroptosis inhibitor (0.5 µM, Fer-1), necrosis inhibitor (20 µM, Nec-1) or apoptosis inhibitor (10 µM z-VAD-fmk), respectively. Calcein-AM / PI double staining was used to detect the survival or death status in each group. (**B**) Quantitative analysis of (**A**). (**C**) Flow cytometry was used to measure cell apoptosis in each group. Q2 and Q3 quadrants are apoptotic cells, and Q1 quadrant contains ferroptosis cells, which can indirectly reflect the degree of ferroptosis. (**D**) Quantitative analysis of (**C**). (**E**) DCFH-DA probe was applied to analyze the ROS content in each group. (**F**) Quantitative analysis of (**E**). (**G**) BODIPY 581/591 C11 probe was applied to analyze the LPO content in each group. (**H**) Quantitative analysis of (**G**). (**I**) MDA level analysis of each group. (**J**) WB analysis for proteins related with apoptosis and ferroptosis in CRC cells. (**K**) Quantitative analysis of (**J**). (‘Nor’ indicates ‘Normoxia’, ‘Hypo’ indicates ‘Hypoxia’, ‘OXA’ indicates ‘Oxaliplatin’, ‘Fer-1’ indicates ‘Ferrostatin-1’, ‘Nec-1’ indicates ‘Necrostatin-1’, ‘z-VAD-fmk’ indicates ‘Apoptosis inhibitor’, ‘OE’ indicates ‘Overexpression’, ‘ASO’ indicates ‘Antisense oligonucleotide’, ‘NC’ indicates ‘Negative control’, ‘Mut’ indicates ‘Mutation’, ‘LPO’ indicates ‘Lipid peroxidation’, ‘ns’ indicates ‘no statistical differences’, ‘*’ indicates ‘P < 0.05’, ‘**’ indicates ‘P < 0.01’, Data are presented as mean ± SD, “N”≥3)

To investigate which cell death pathway is responsible for the sensitization of oxaliplatin by ferrous iron (LIP) accumulation, ferroptosis inhibitor (Fer-1), necrosis inhibitor (Nec-1) and apoptosis inhibitor (z-VAD-fmk) were added to normoxic CRC cells, respectively. As shown in Fig. 6A and B, the addition of Fer-1 or z-VAD-fmk alone reduced cell death, which was statistically significant. Moreover, the simultaneous addition of Fer-1 and z-VAD-fmk almost completely reversed the cell death induced by oxaliplatin combined with iron. However, Nec-1 did not affect the percentage of PI-positive cells. These results preliminarily indicated that LIP accumulated in CRC cells promoted the killing effect of oxaliplatin combined with iron by inducing apoptosis and ferroptosis.

The apoptosis of each group was detected by flow cytometry (five groups as follow: OE NC, OE lnc-IRP, OE lnc-IRP Mut, ASO NC, ASO lnc-IRP). The results showed that overexpression of lnc-IRP (but not lnc-IRP Mut) resulted in less apoptosis, while knockdown of lnc-IRP reversed oxaliplatin resistance induced by hypoxia (Fig. 6C and D). Meanwhile, the contents of reactive oxygen species (ROS), lipid peroxidation (LPO), malondialdehyde (MDA), reduced glutathione (GSH) and lutathione peroxidase 4 (GPX4) activity were detected. The results showed that under normoxic culture conditions, the levels of ROS, LPO and MDA were decreased, whereas GSH levels and GPX4 activity were elevated in the lnc‑IRP‑overexpressing (OE lnc‑IRP) group. However, no significant alterations were observed in the lnc‑IRP‑mutant‑overexpressing (OE lnc‑IRP Mut) group. Under hypoxic conditions, ROS, LPO and MDA levels were increased, while GSH levels and GPX4 activity were reduced in the lnc‑IRP‑knockdown (ASO lnc‑IRP) group (Fig. 6E and I, Fig. S1G- S1H). These results suggested that lower sensitivity to oxaliplatin in CRC induced by the lnc-IRP / IRP2 / LIP pathway is accompanied by a blocked ferroptosis. The expressions of apoptosis-related (caspase3, cle-caspase3, bcl2, bax) and ferroptosis-related (PTGS, FTH1, GPX4) proteins in the above groups were detected for further verification (Fig. 6J and K, Fig. S1I-S1J). After overexpression of lnc-IRP that contains complete IRE-like elements in normoxic condition, the proteins associated with activation of apoptosis and ferroptosis were declined (cle-Caspase3, Bax, PTGS2), while the negative regulatory proteins were augmented (Caspase3, Bcl2, FTH1, GPX4). In contrast, after knocking down the lnc-IRP in hypoxic CRC, the expressions of cle-Caspase3, Bax and PTGS2 were raised, while Caspase3, Bcl2, FTH1, and GPX4 were diminished.

In summary, it has been proved in vitro that hypoxia-associated lnc-IRP, which relies on IRE-like elements to competitively bind IRP2, interferes with iron homeostasis, resulting in shrunk LIP accumulation, reduced ROS formation, blocked apoptosis and ferroptosis, and ultimately weakened the sensitivity to oxaliplatin of CRC.

### Hypoxia-driven lnc-IRP / IRP2 / iron-metabolism axis was further confirmed to suppress oxaliplatin sensitivity of CRC in vivo

**Fig. 7:**
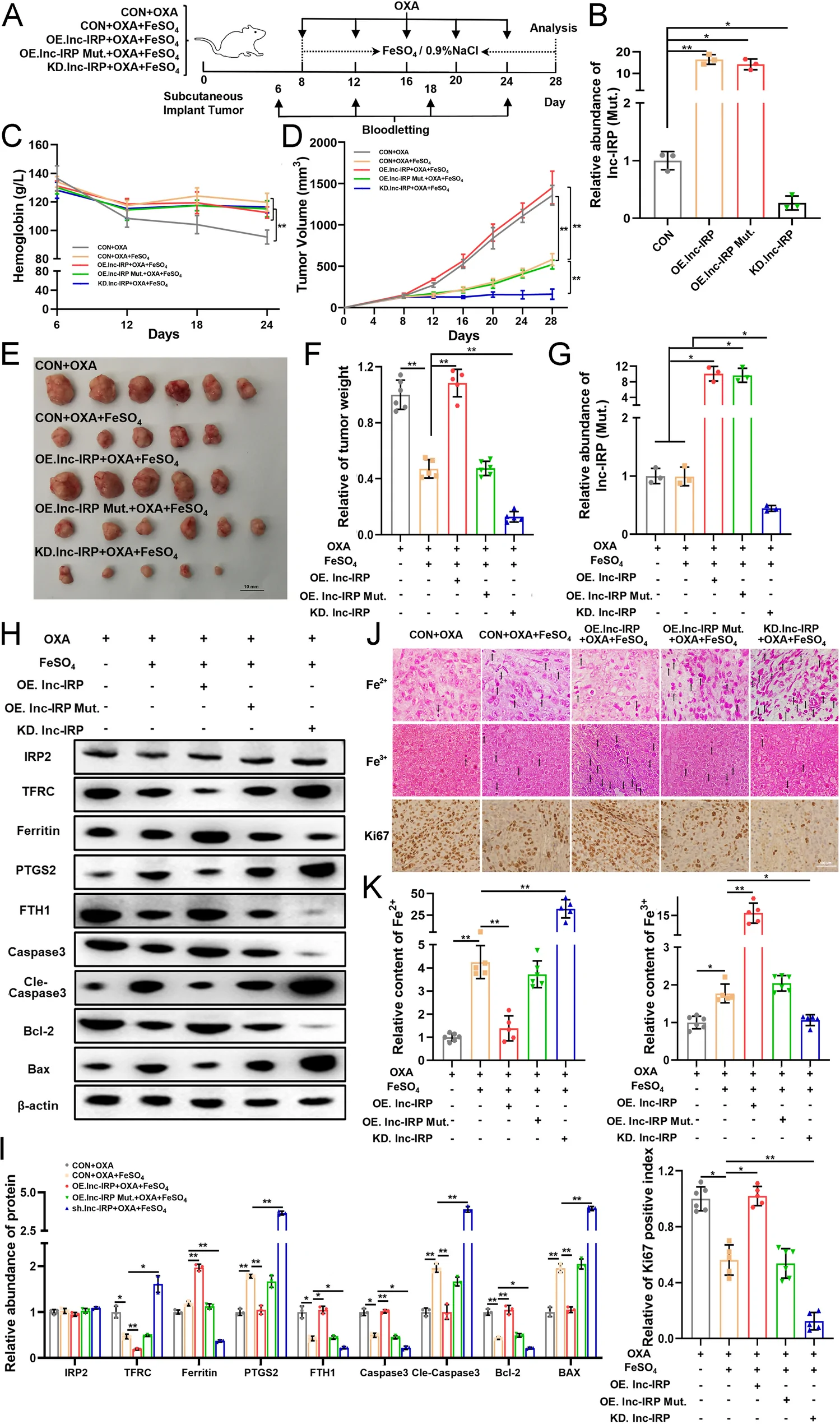
In *vivo*, lnc-IRP with an intact IRE-like element suppresses apoptosis and ferroptosis by disrupting iron metabolism and ultimately reduces the sensitivity of CRC to oxaliplatin. (**A**) A simplified flowchart of the animal experiment. (**B**) PCR analysis of HCT116 cell that infected with lentivirus. (**C**) ELISA assay was performed to evaluate the amount of hemoglobin in mice serum. (**D**) Volume changes of transplanted tumors during tumor-bearing period. (**E**) Pictures of the tumors in all groups. (**F**) Weight of the tumors. (**G**) RT–qPCR quantification of lnc-IRP or lnc-IRP Mut within the tumors. (**H**) WB analysis for proteins related with iron-metabolism, apoptosis and ferroptosis within the tumors. (**I**) Quantitative analysis of (**H**). (**J**) Turnbull’s blue reaction for ferrous iron analysis, Perls’ blue reaction for ferric iron analysis, and IHC of Ki67 for proliferation detection. (**K**) Quantitative analysis of (**J**). (‘OXA’ indicates ‘Oxaliplatin’, ‘OE’ indicates ‘Overexpression’, ‘KD’ indicates ‘Knock down’, ‘CON’ indicates ‘Control’, ‘Mut’ indicates ‘Mutation’, ‘IHC’ indicates ‘Immunohistochemistry’, ‘*’ indicates ‘P < 0.05’, ‘**’ indicates ‘P < 0.01’, Data are presented as mean ± SD, “N” ≥3)

**Fig. 8:**
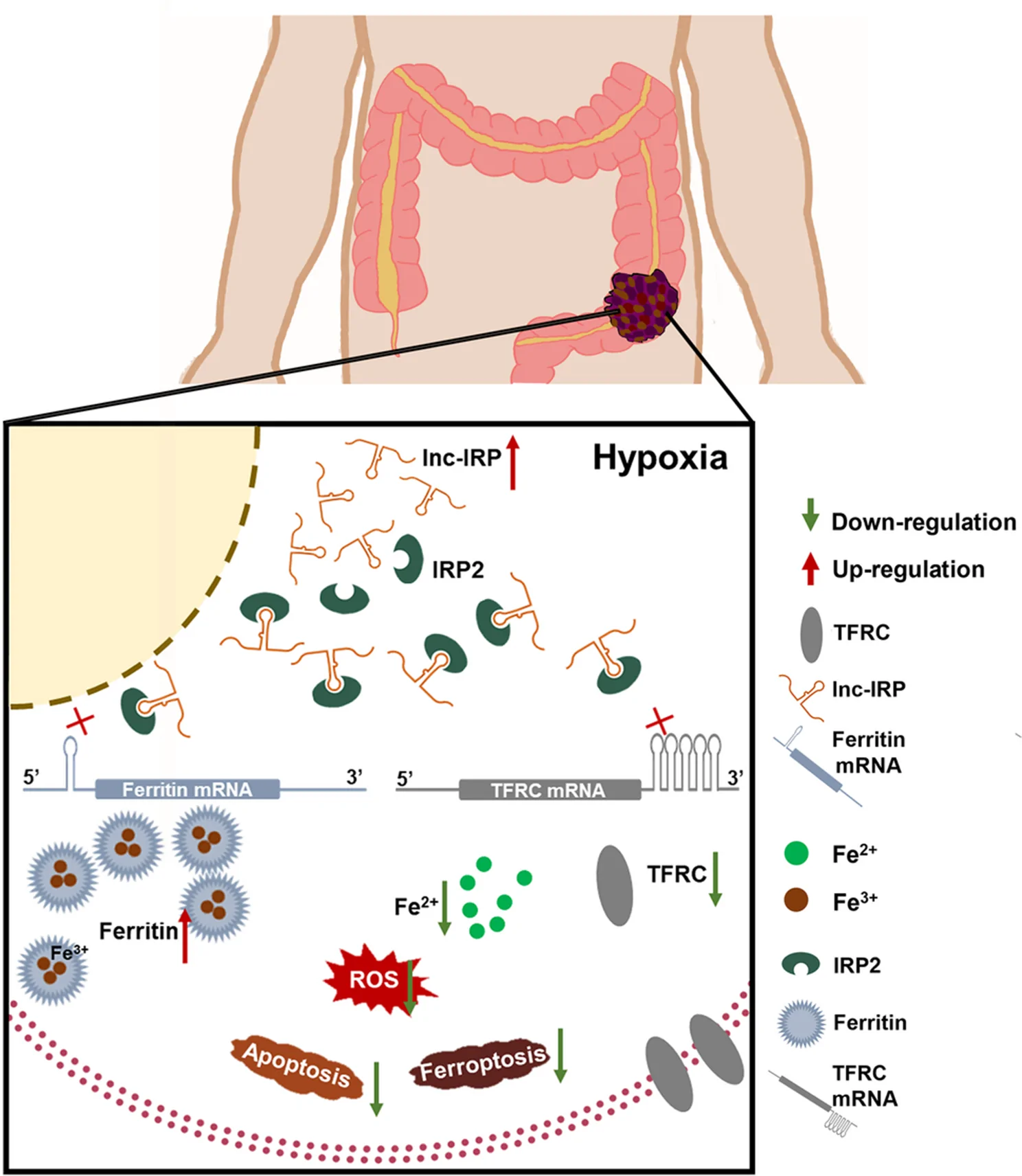
A proposed mechanistic model in this study

Figure 7A is a simplified flowchart of the animal experiment. HCT116 cells were infected with negative control (CON) lentivirus, lnc-IRP overexpressing lentivirus (OE lnc-IRP), lnc-IRP-Mut overexpressing lentivirus (OE lnc-IRP Mut), and lnc-IRP knockdown lentivirus (KD lnc-IRP), respectively (Fig. 7B). Subsequently, a xenograft tumor model of nude mice was established (*n* = 30). There were five groups in total, model control group, control group, lnc-IRP overexpressing group, lnc-IRP Mut overexpressing group and lnc-IRP knockdown group. The first group was treated with oxaliplatin only, and the last four groups were treated with oxaliplatin combined with iron supplement. Caudal vein bloodletting was performed from day sixth, 0.01mL/g each time, once every six day. The hemoglobin of mice was detected by ELISA. Results revealed that it was reduced with bloodletting in all groups. Except the model control group, the hemoglobin levels were stabilized and did not further decrease in all groups receiving iron supplementation (Fig. 7C). During the tumor-bearing period, tumor growth was recorded (Fig. 7D). After 28 days, subcutaneous grafts were harvested surgically (Fig. 7E). All transplanted tumors were weighed (Fig. 7F). The changes of lnc-IRP or lnc-IRP Mut in tumor tissues were confirmed by RT-qPCR (Fig. 7G). The changes of proteins related to iron metabolism, apoptosis and ferroptosis in transplanted tumor tissues were measured (Fig. 7H and I). In addition, the content of ferrous and ferric iron was detected by the Turnbull’s blue reaction and Perls’ blue reaction, respectively. Moreover, proliferation was analyzed by immunohistochemistry (IHC) of Ki67 (Fig. 7J and K). The results further demonstrated that lnc-IRP was dependent on its IRE-like elements to break iron homeostasis, inhibit apoptosis and ferroptosis, and thus reduce the sensitivity of CRC to oxaliplatin. It also suggests that targeted down-regulation of lnc-IRP is expected to reverse the reprogramming of iron metabolism induced by hypoxia and ultimately boost drug efficacy (Fig. 8).

## Discussion

The effect of iron on tumor can be roughly summarized into two aspects. On the one hand, from the perspective of epidemiology, the correlation between iron intake or iron-related indicators such as serum ferritin and tumor formation was analyzed. Previous studies have shown that there is not a simple positive or negative correlation between the two mentioned above, but a complex and diverse correlation in various tumors [34–37]. However, due to differences in the participants and analysis methods, inconsistent conclusions are presented even for the same cancer. On the other hand, the effect of iron on the biological function of tumor was analyzed. ROS induced by ferrous iron can be regarded as a therapeutic target to kill tumors by enhancing intracellular oxidative stress [38]. Studies have found that bicalutamide combined with iron can increase the effect of anti-androgen therapy for prostate cancer by aggravating oxidative damage [39]. The production of ROS in situ by Fenton reaction or Fenton-like reaction is the theoretical basis of chemodynamic therapy (CDT). Based on this, iron-containing nanoparticles may inhibit tumor progression by triggering harmful oxidative stress. At present, the development and improvement of biodegradable and efficient materials is in progress [40].

CRC patients are often accompanied by gastrointestinal bleeding, so there is a basis of iron homeostasis imbalance [2, 10, 11]. Previous investigations have confirmed that iron deficiency is associated with poor prognosis of CRC [41–43]. Red blood cell (RBC) transfusion and erythropoiesis-stimulating agents are associated with an increased risk of recurrence and death, so direct iron supplementation is the preferred method for iron deficiency in CRC [44–46]. However, whether iron supplementation can synergistically enhance the effect of chemotherapy remains unclear. This study focused on the role of iron in oxaliplatin chemotherapy of CRC. It was found that iron supplementation can successfully up-regulate ferrous iron in normoxic CRC cells, thus promoting the toxic effect of oxaliplatin. This is consistent with the above theory that LIP can destroy tumors by inducing oxidation and antioxidant imbalance. However, in hypoxic CRC cells, supplemental iron is not effectively converted into LIP. The results indicate that iron metabolism is reprogrammed at the post-translational level of IRP2 under oxygen-starved conditions. It is reasonable to hypothesize that the IRE binding site of IRP2 may be occupied by other RNAs that contain IRE-like elements in hypoxic CRC.

LncRNAs are mainly involved in tumorigenesis and progression by three means. Firstly, lncRNAs can act as endogenous competing RNAs (ceRNAs), which rely on the principle of base complementary pairing to mediate the interactions between RNAs [47]. Secondly, with the development of ribosome profiling sequencing, recent studies have found that some lncRNAs have the ability to encode small peptides [48]. Thirdly, it can function by combining with RBP, and this study is based on this mechanism. LncRNA, as a linear RNA with a longer sequence, is more likely to have a local secondary structure similar to mRNA. Therefore, the hypothesis above can be further refined, that is, lncRNA containing IRE-like elements may play a role as interfering molecules between IRP2 and its downstream target in hypoxic CRC. And lnc-IRP was screened by the combination of RIP-seq and lncRNA seq. Different from previous studies on lncRNA-protein interactions, this study individually targeted the screening of lnc-IRP containing specific secondary structures based on the sequence and structural characteristics of IRE elements, with clearer purpose and novel perspectives.

The efficient transport of oxygen throughout the body requires the involvement of iron. Hypoxia induces the synthesis of erythropoietin in the kidney and inhibits the production of hepcidin in the liver, thereby mobilizing the absorption and utilization of iron and promoting the generation of hemoglobin and erythrocytes. At the cellular level, hypoxic microenvironment can regulate the expression of genes related to iron metabolism. Studies has shown that FBXL5-mediated degradation of IRP2 by ubiquitination is inhibited under hypoxia conditions [49]. This provides a possible explanation for the upregulation of IRP2 expression in hypoxic CRC observed in this study.

RNA-based therapeutics mainly include antisense oligonucleotides (ASO), small interfering RNA (siRNA), short hairpin RNA (shRNA), ASO anti-miRNA (antimiRs), miRNA mimics, miRNA adsorption sponges, therapeutic circRNA, and CRISPR-Cas9-based gene editing technology [50]. At present, there are more than ten RNA therapeutics, mainly belonging to siRNA or ASO and including mRNA vaccines for COVID-19, that have been approved by the FDA or the European Medicine Agency (EMA). But there is no approval in oncology. lncRNA-based therapeutics started later than miRNA and gradually became a hot topic of research in the past decade. Although the complex function of lncRNA increases the difficulty of study, it also brings multiple possibilities of targeted therapy and a vast application prospect. This study confirmed that targeting down-regulation of lnc-IRP by ASO could reverse the hypoxia-induced iron metabolic reprogramming at the post-translational level of IRP2, thereby enhancing the sensitivity of CRC to oxaliplatin. Therefore, lnc-IRP is expected to be a new breakthrough in lncRNA therapeutics.

## Conclusion

This study reported that upregulation of lnc-IRP sequesters apoptosis and ferroptosis via interfering with the function of IRP2 by its IRE-like element and curtails the production of LIP and the accumulation of ROS, thereby triggering drug resistance of CRC. Targeted blockade of lnc-IRP can reverse the imbalanced iron homeostasis induced by hypoxic microenvironment and increase the chemosensitivity of CRC. This work deciphers a novel molecular mechanism that hypoxia-related reprogramming of iron metabolism participates in the regulation of chemotherapy sensitivity, based on which this work offers a promising therapeutic approach for clinical trials.

## Supplementary Information

Below is the link to the electronic supplementary material.


Supplementary Material 1



Supplementary Material 2



Supplementary Material 3



Supplementary Material 4


## Data Availability

The datasets generated during and/or analyzed during the current study are available from the corresponding author upon reasonable request.
