# Supplementary material for: Hypoxia-induced lnc-IRP drives oxaliplatin resistance in colorectal cancer through IRP2 sequestration and iron metabolism reprogramming
Source: J Transl Med. 2026 Jun 22;24:1120. doi: 10.1186/s12967-026-08440-3 (PMC13528089; doi:10.1186/s12967-026-08440-3)
Supplement: Supplementary file 1 [file 12967_2026_8440_MOESM3_ESM.pdf]

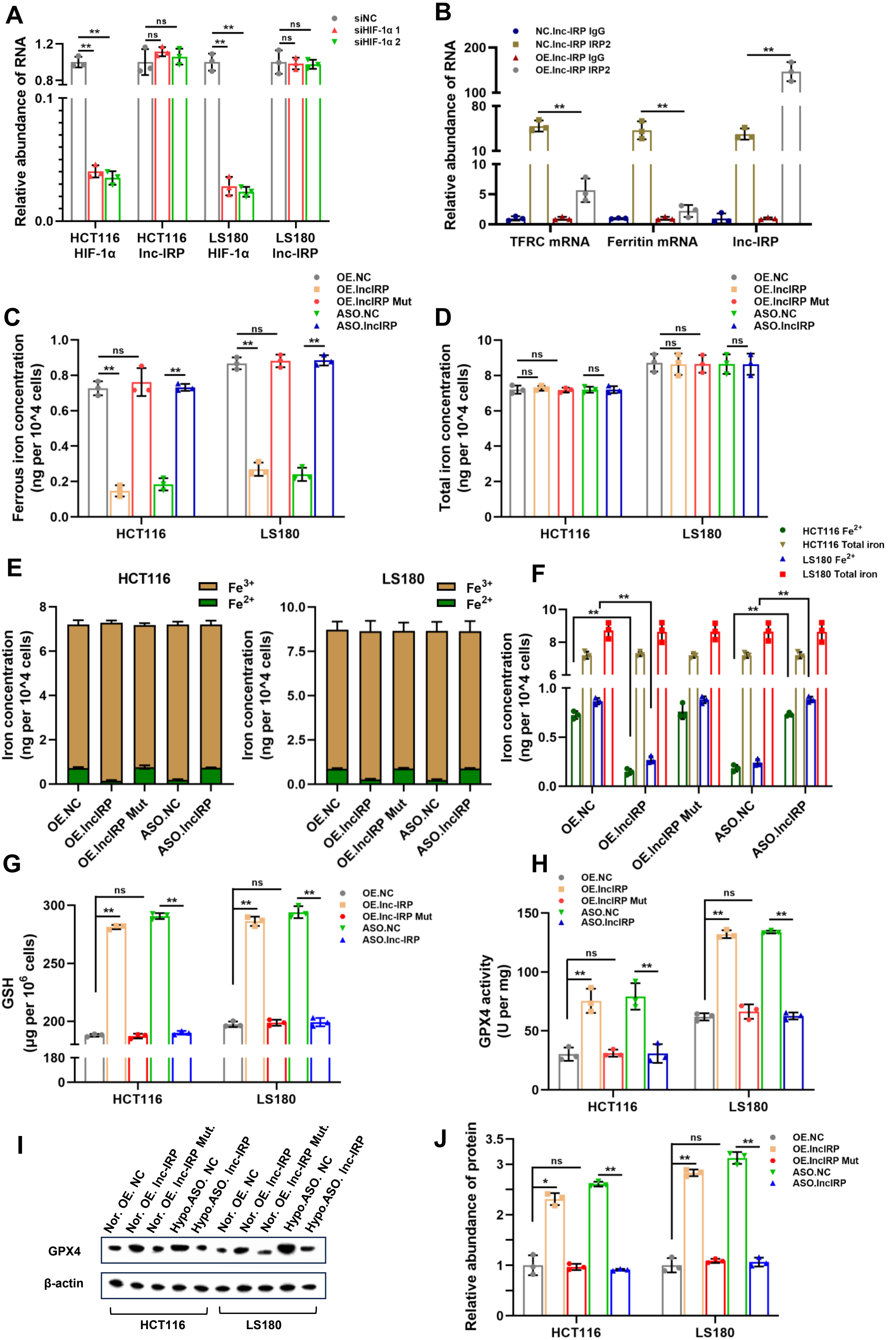
A
Relative abundance of RNA
siNC
siHIF-1α 1
siHIF-1α 2
HCT116 HIF-1α
HCT116 lnc-IRP
LS180 HIF-1α
LS180 lnc-IRP
B
Relative abundance of RNA
NC.lnc-IRP IgG
NC.lnc-IRP IRP2
OE.lnc-IRP IgG
OE.lnc-IRP IRP2
TFRC mRNA
Ferritin mRNA
lnc-IRP
C
Ferrous iron concentration (ng per 10^4 cells)
OE.NC
OE.lncIRP
OE.lncIRP Mut
ASO.NC
ASO.lncIRP
HCT116
LS180
D
Total iron concentration (ng per 10^4 cells)
E
HCT116
LS180
Fe3+
Fe2+
Iron concentration (ng per 10^4 cells)
F
HCT116 Fe2+
HCT116 Total iron
LS180 Fe2+
LS180 Total iron
G
GSH (μg per 10^6 cells)
OE.Inc-IRP
OE.Inc-IRP Mut
ASO.Inc-IRP
H
GPX4 activity (U per mg)
I
Nor. OE. NC
Nor. OE. lnc-IRP
Nor. OE. lnc-IRP Mut.
Hypo.ASO. NC
Hypo.ASO. lnc-IRP
GPX4
β-actin
J
Relative abundance of protein

Figure legend S1

(A) Hypoxia-induced upregulation of lnc-IRP is largely independent of HIF-1α as verified by siRNA-mediated HIF-1α knockdown followed by qRT-PCR detection. (B) RNA-RIP-qPCR assays demonstrated that lnc-IRP competitively bound to IRP2 and displaced IRP2 from its canonical downstream target TFRC and ferritin mRNAs. (C) Bar plot of individual intracellular ferrous iron (Fe^{2+}) levels across five groups. (D) Bar plot of individual intracellular total iron levels across five groups. (E) Stacked bar graph illustrating the proportional distribution of ferrous iron (Fe^{2+}) and ferric/bound iron (Fe^{3+}, calculated by total iron minus Fe^{2+}) in intracellular iron pools. (F) Grouped bar graph showing parallel changes of Fe^{2+} and total iron in all treatment groups. (G) Intracellular GSH content. (H) GPX4 enzymatic activity. (I) Representative Western blot of GPX4 protein. (J) Quantification of GPX4 protein levels. All data are presented as mean ± SD from three independent biological replicates. Statistical significance was determined by two-tailed unpaired Student's t-test. $*p < 0.05$, $**p < 0.01$; ns, no significant.
