# Supplementary material for: Hypoxia-induced lnc-IRP drives oxaliplatin resistance in colorectal cancer through IRP2 sequestration and iron metabolism reprogramming
Source: J Transl Med. 2026 Jun 22;24:1120. doi: 10.1186/s12967-026-08440-3 (PMC13528089; doi:10.1186/s12967-026-08440-3)

Fig 1I

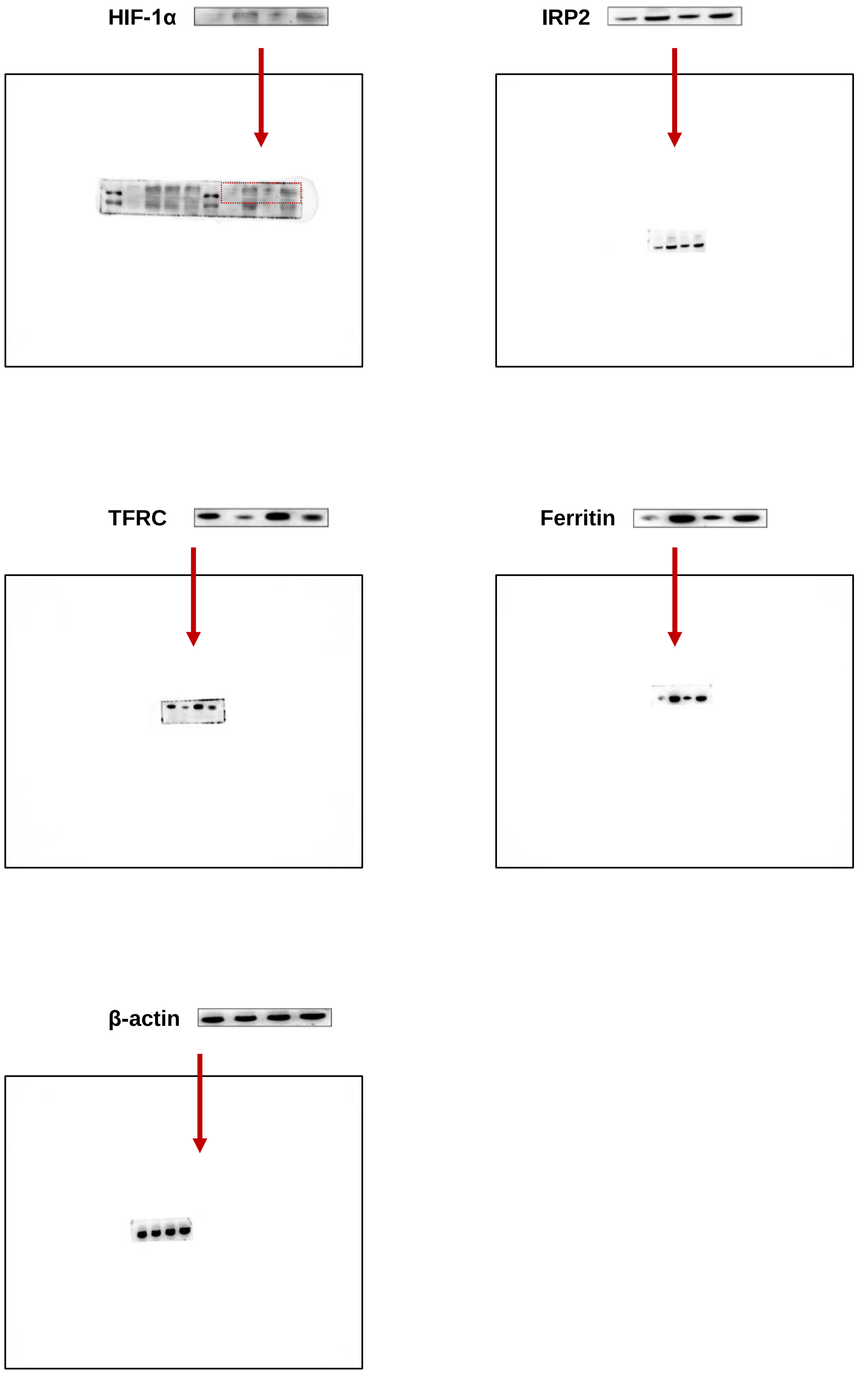

Fig 4A

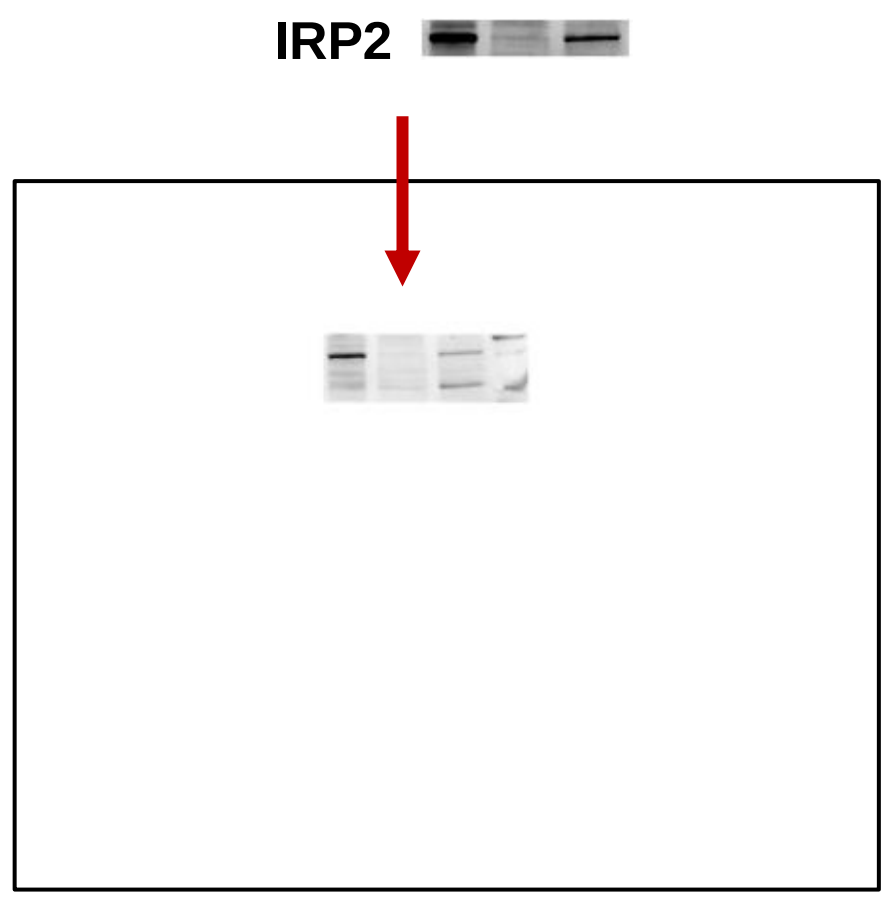
IRP2

Fig 4F

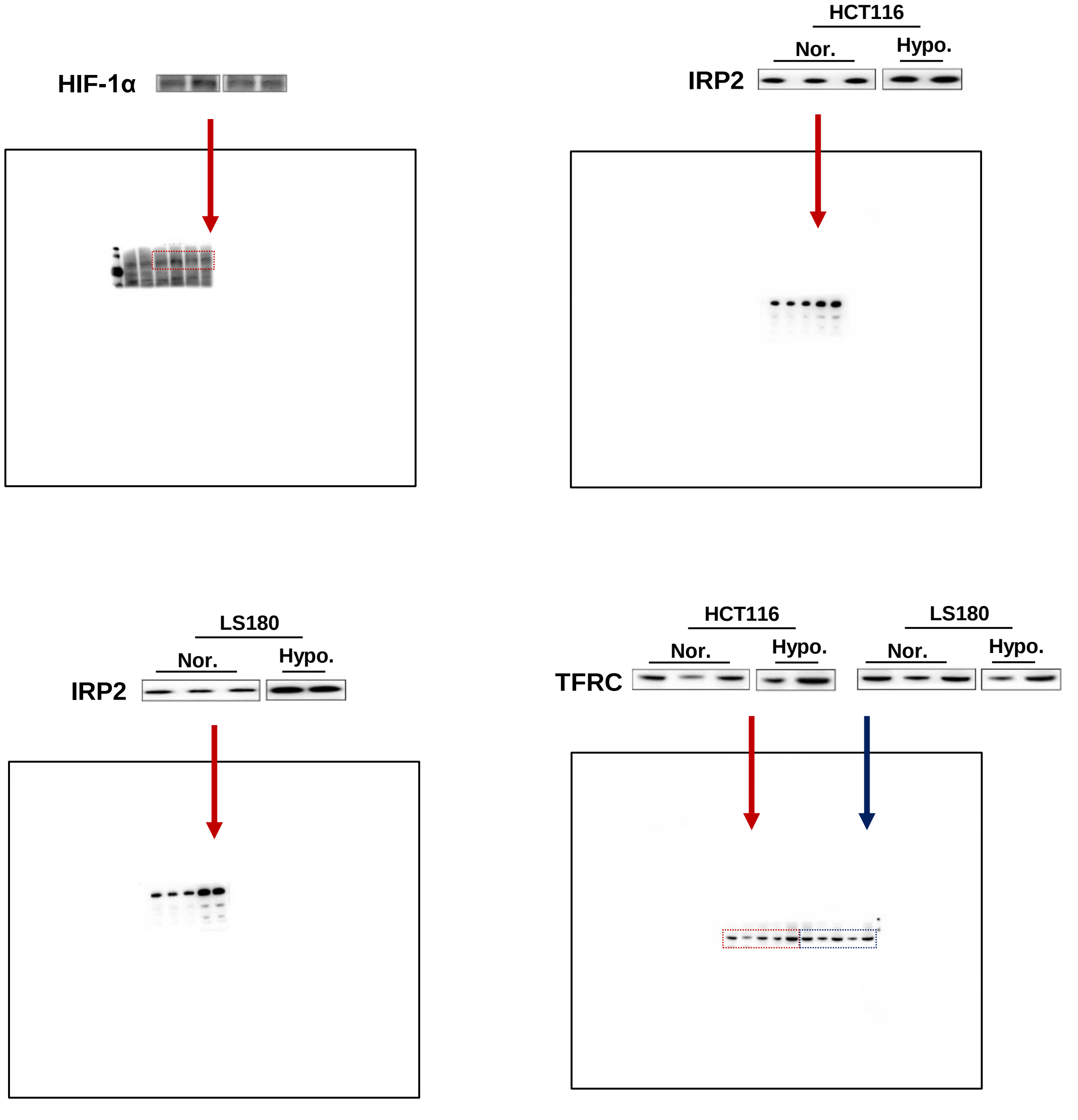
HIF-1α
HCT116
Nor.
Hypo.
IRP2
LS180
Nor.
Hypo.
IRP2
HCT116
Nor.
Hypo.
LS180
Nor.
Hypo.
TFRC

Fig 4F

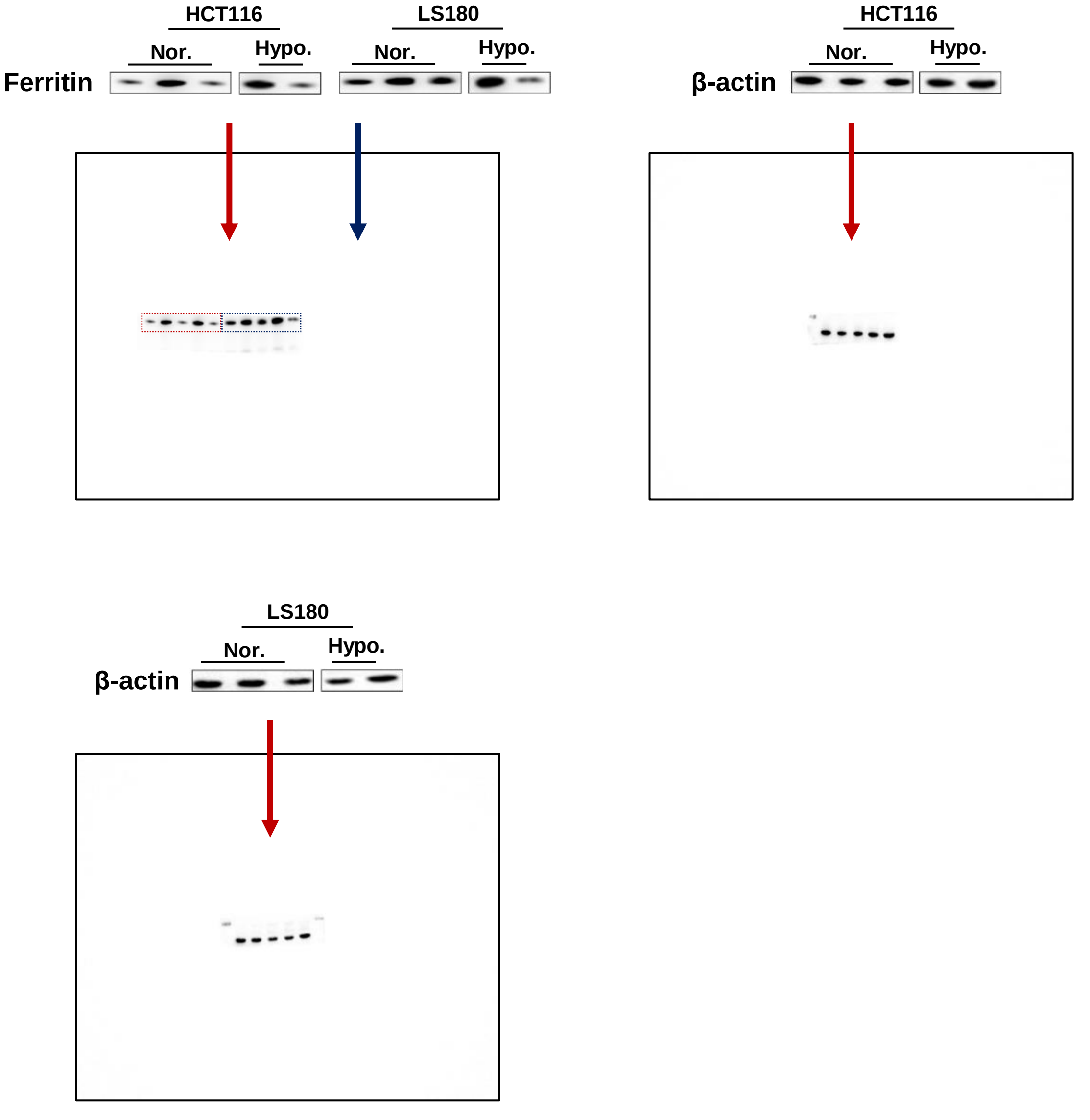
HCT116
Nor.
Hypo.
LS180
Nor.
Hypo.
Ferritin
HCT116
Nor.
Hypo.
β-actin
LS180
Nor.
Hypo.
β-actin

Fig 6J

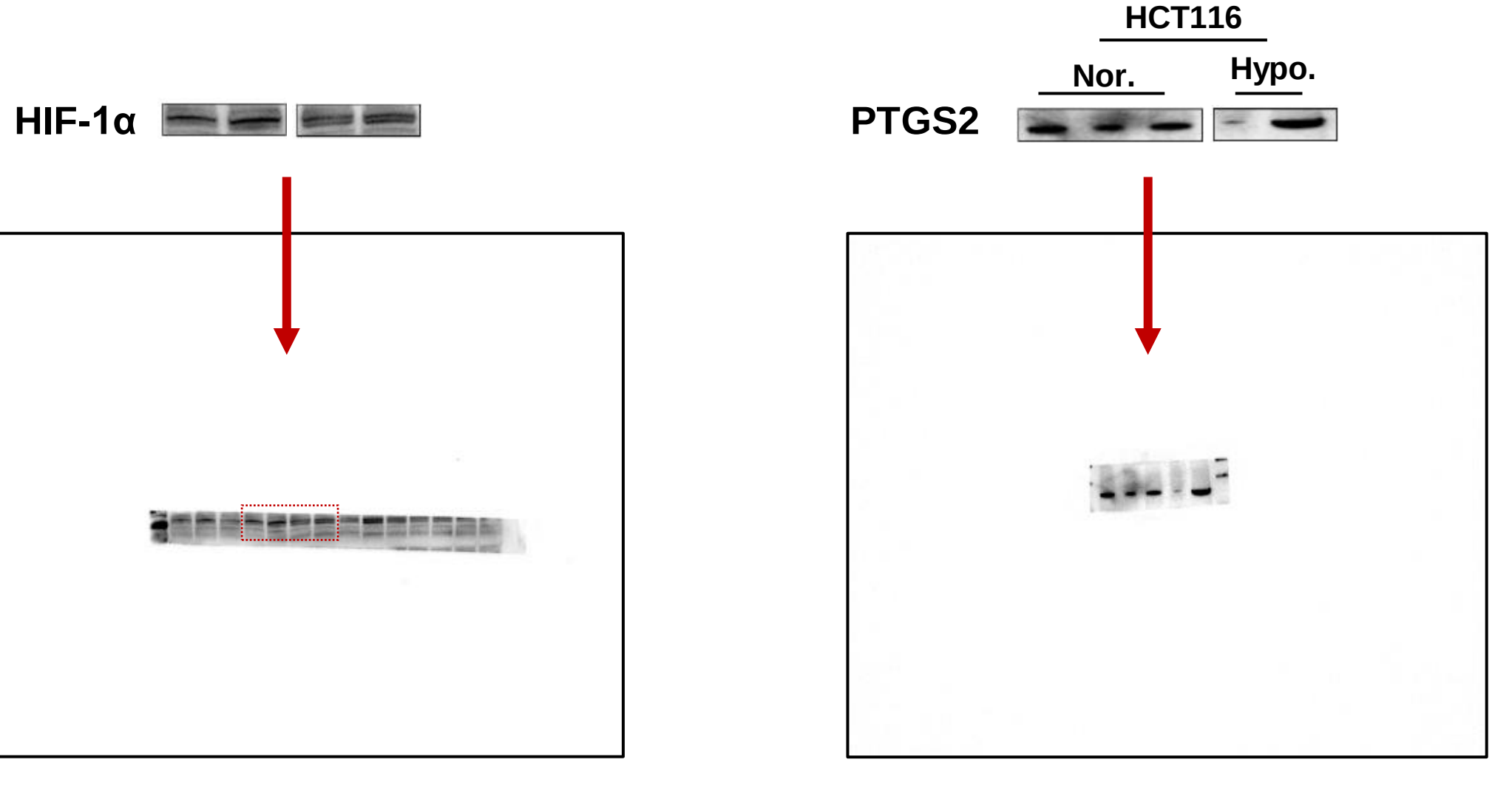
HIF-1α
HCT116
Nor.
Hypo.
PTGS2

Fig 6J

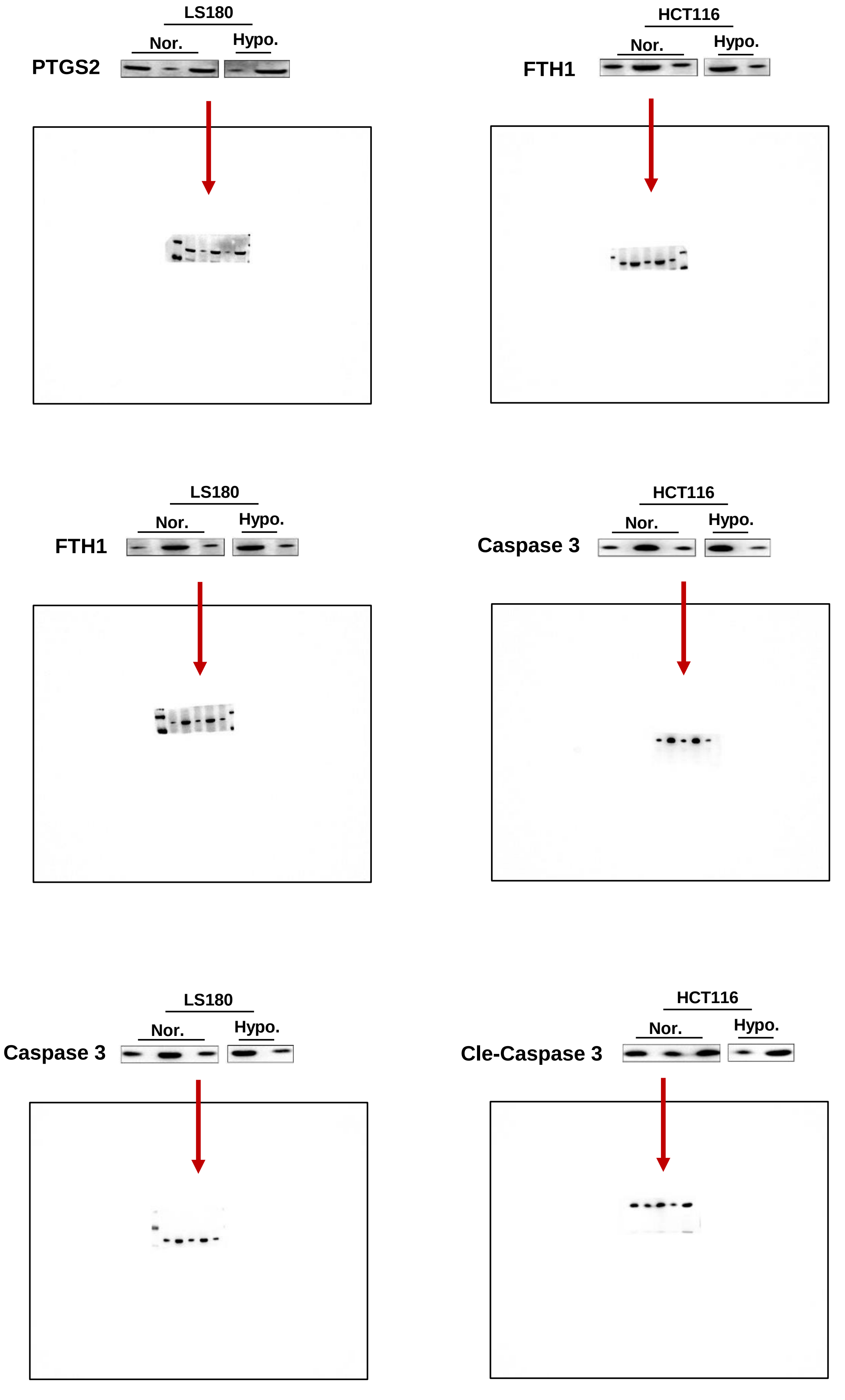

LS180
Nor.
Hypo.
PTGS2
HCT116
Nor.
Hypo.
FTH1
LS180
Nor.
Hypo.
FTH1
HCT116
Nor.
Hypo.
Caspase 3
LS180
Nor.
Hypo.
Caspase 3
HCT116
Nor.
Hypo.
Cle-Caspase 3

Fig 6J

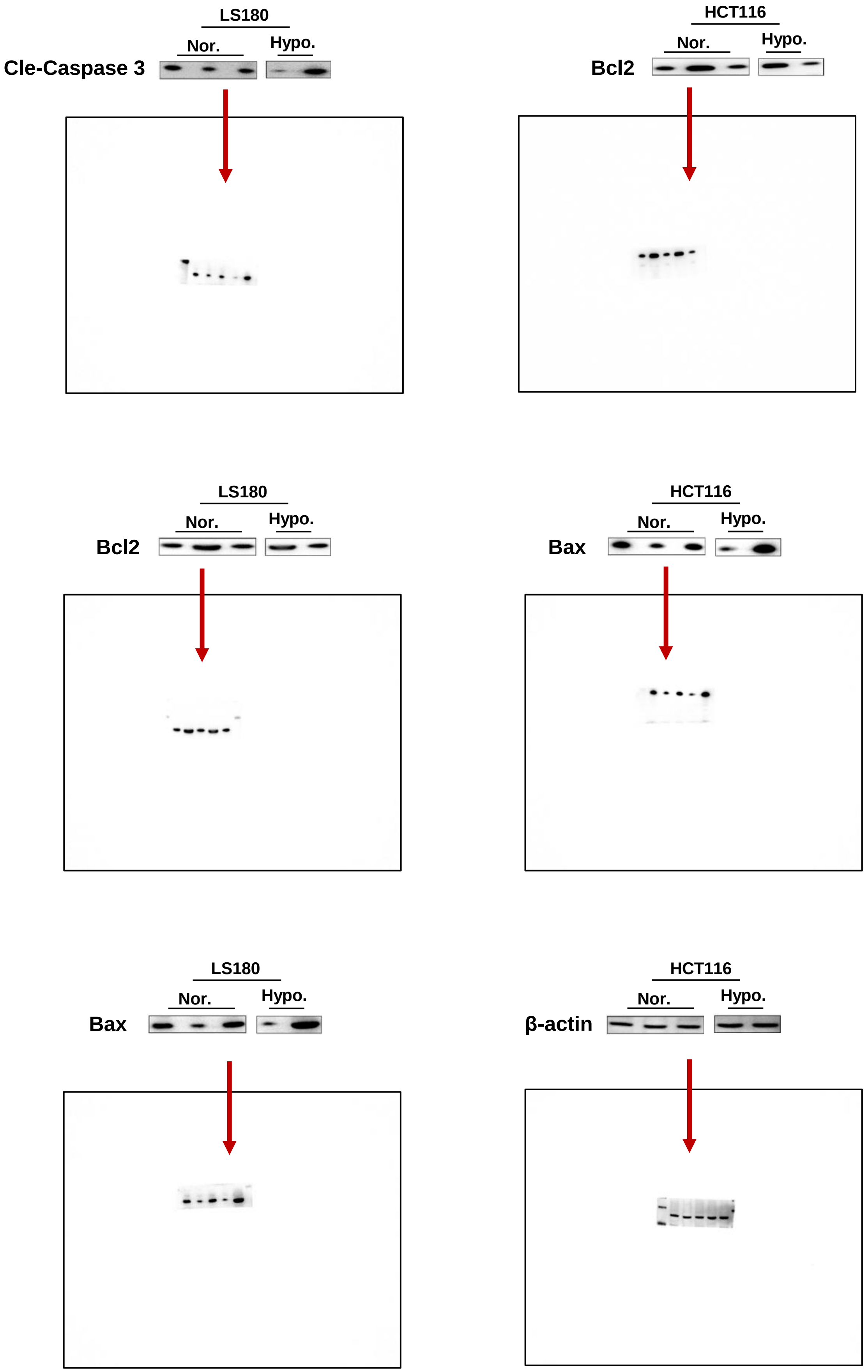
LS180
Nor.
Hypo.
Cle-Caspase 3
HCT116
Nor.
Hypo.
Bcl2
LS180
Nor.
Hypo.
Bcl2
HCT116
Nor.
Hypo.
Bax
LS180
Nor.
Hypo.
Bax
HCT116
Nor.
Hypo.
β-actin

Fig 6J

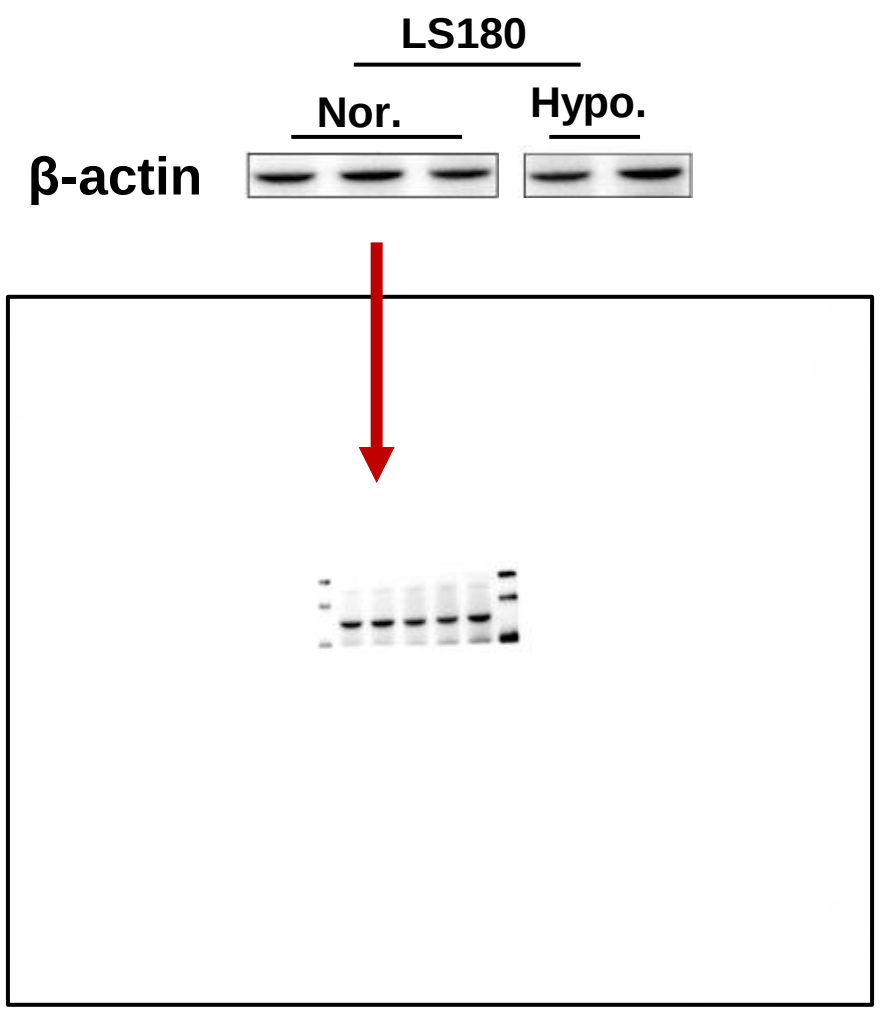

Fig 7H

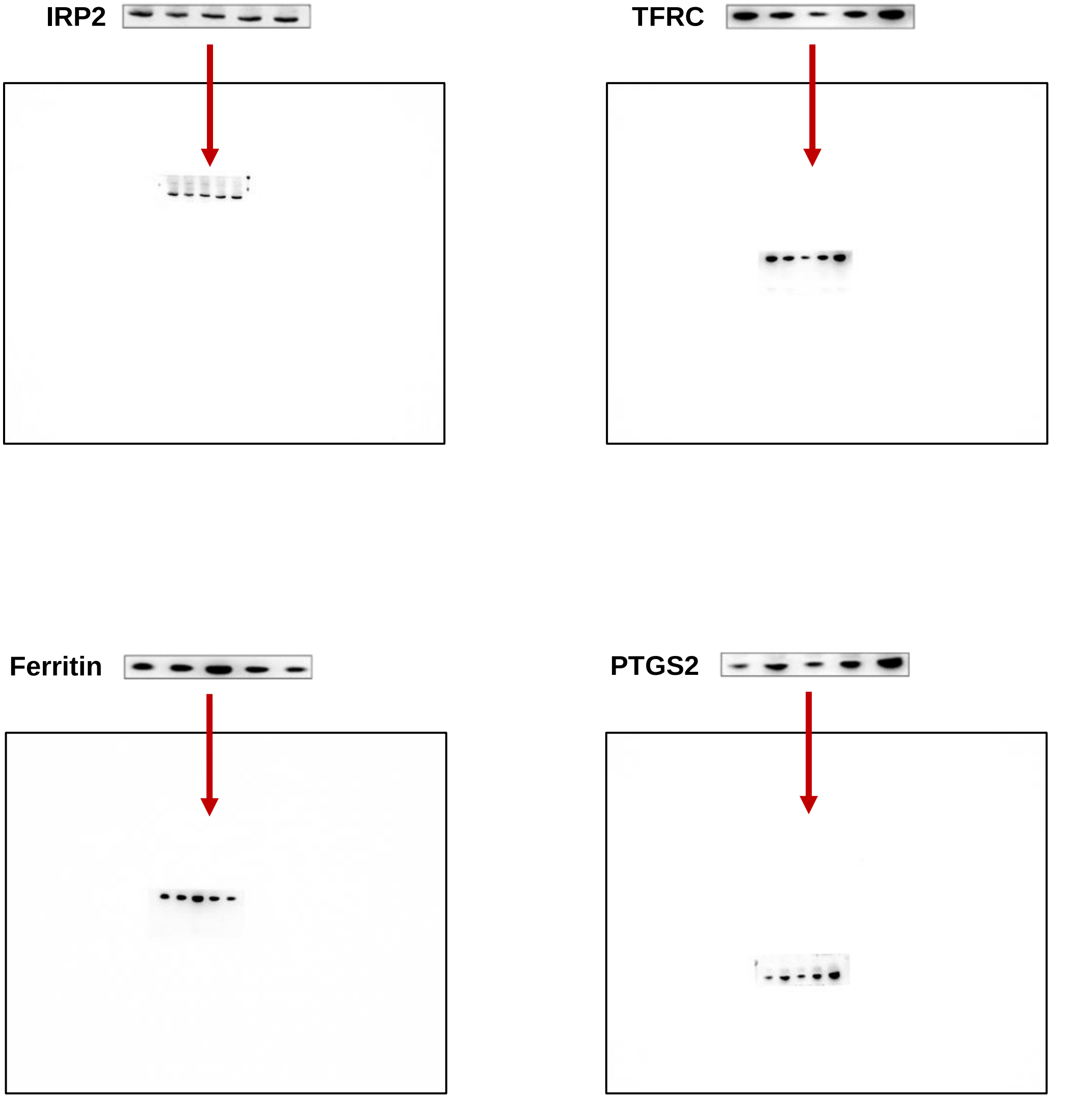

Fig 7H

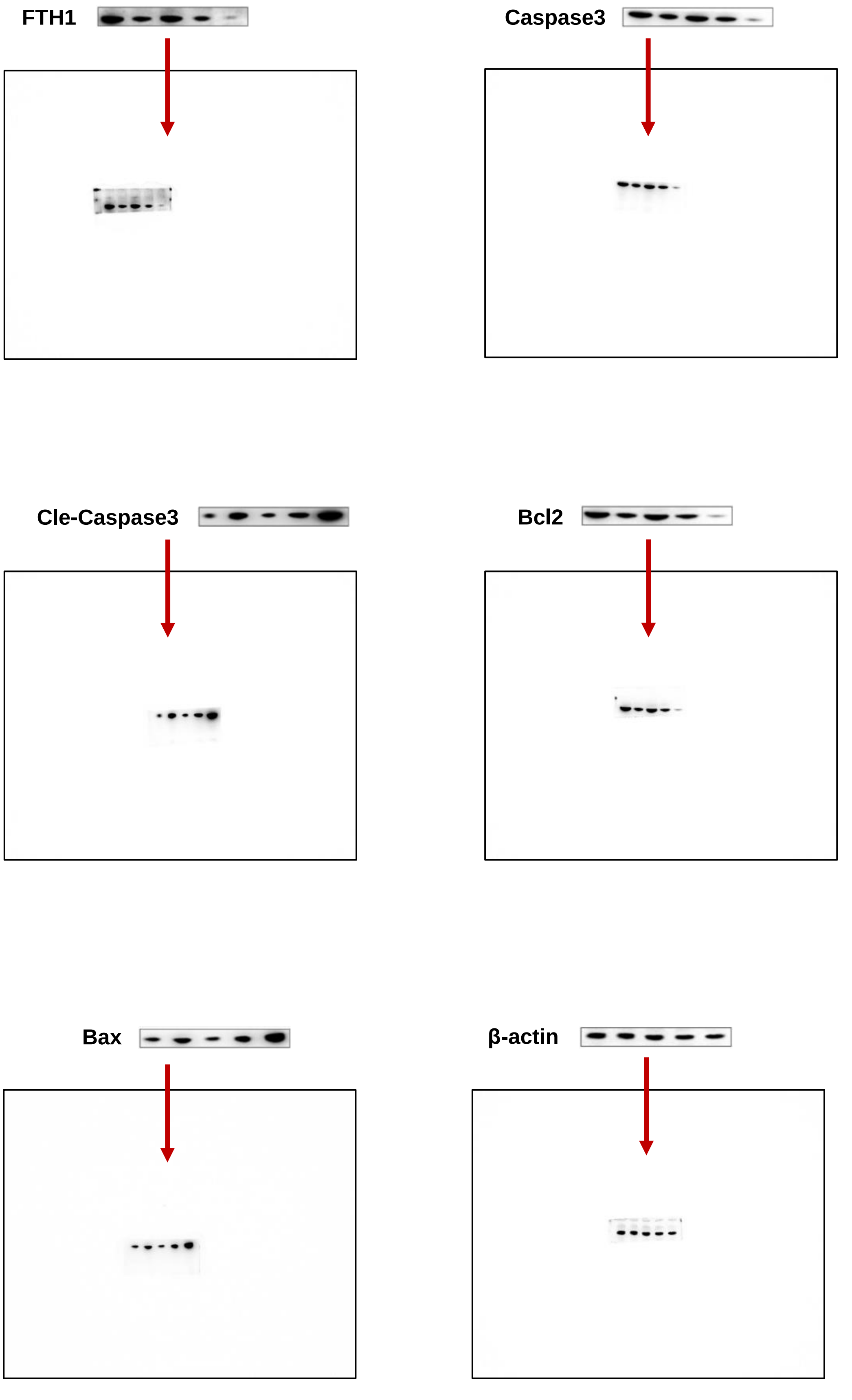

Fig S1I

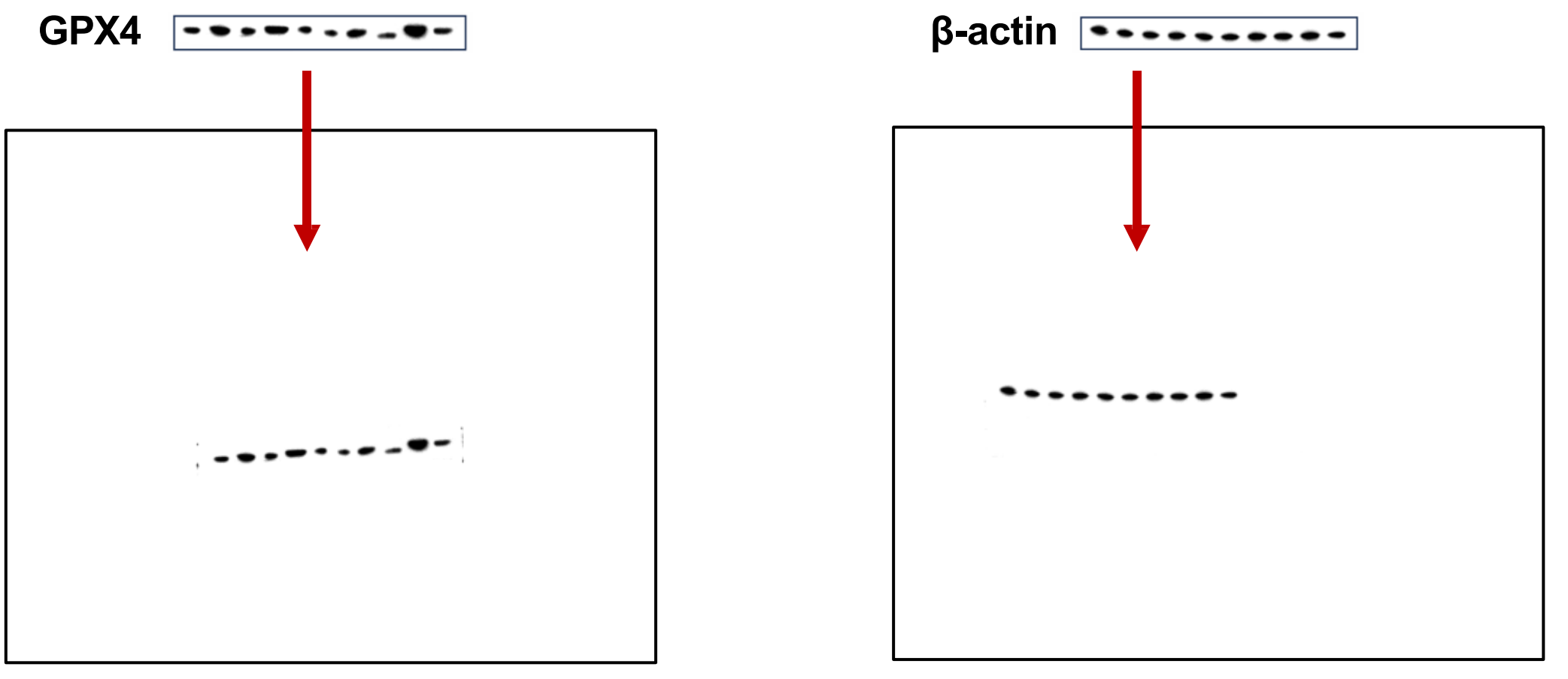

Supplement: Supplementary file 2 [file 12967_2026_8440_MOESM4_ESM.pdf]
