# Supplementary material for: Hypoxia-induced lnc-IRP drives oxaliplatin resistance in colorectal cancer through IRP2 sequestration and iron metabolism reprogramming
Source: J Transl Med. 2026 Jun 22;24:1120. doi: 10.1186/s12967-026-08440-3 (PMC13528089; doi:10.1186/s12967-026-08440-3)
Supplement: Supplementary file 3 [file 12967_2026_8440_MOESM1_ESM.docx]

**Supplementary Material**

**Table S1**

The relative expression of lnc-IRP and the relative content of total iron in CRC tissue

| Characteristic | N | lnc-IRP | | |  | Total Iron | |
| --- | --- | --- | --- | --- | --- | --- | --- |
|  |  | $\bar{x}\pm SD$ | P value | |  | $\bar{x}\pm SD$ | P value |
| Gender |  |  | | 0.394 |  |  | 0.934 |
| Male | 55 | 1.04± 0.46 | |  |  | 1.00± 0.42 |  |
| Female | 47 | 0.96± 0.54 | |  |  | 0.99± 0.38 |  |
| Age |  |  | | 0.536 |  |  | 0.476 |
| < 60 | 48 | 1.12± 0.51 | |  |  | 0.90± 0.38 |  |
| ≥ 60 | 54 | 0.90± 0.48 | |  |  | 1.09± 0.40 |  |
| Location |  |  | | 0.392 |  |  | 0.466 |
| Colon | 88 | 1.02± 0.49 | |  |  | 0.99± 0.40 |  |
| Rectosigmoid junction | 3 | 0.64± 0.29 | |  |  | 0.87± 0.22 |  |
| Rectum | 11 | 0.93± 0.60 | |  |  | 1.13± 0.47 |  |
