# Supplementary material for: Hypoxia-induced lnc-IRP drives oxaliplatin resistance in colorectal cancer through IRP2 sequestration and iron metabolism reprogramming
Source: J Transl Med. 2026 Jun 22;24:1120. doi: 10.1186/s12967-026-08440-3 (PMC13528089; doi:10.1186/s12967-026-08440-3)
Supplement: Supplementary file 4 [file 12967_2026_8440_MOESM2_ESM.docx]

**Table S2**

Univariate and multivariate Cox regression analyses for progression-free survival

| Variables | Univariate analysis |  | Multivariate Model 1 (Core model) |  | Multivariate Model 2  (Full adjusted model) |  |
| --- | --- | --- | --- | --- | --- | --- |
|  | HR (95%CI) | P value | HR (95%CI) | P value | HR (95%CI) | P value |
| Iron metabolism indexes |  |  |  |  |  |  |
| Total iron | 0.983(0.945-1.023) | 0.411 | — | — | — | — |
| Fe^2+^ | 0.464(0.320-0.673) | ＜0.001 | 0.434(0.333-0.567) | ＜0.001 | 0.426(0.322-0.564) | ＜0.001 |
| Fe^3+^ | 0.948(0.698-1.287) | 0.733 | — | — | — | — |
| lnc-IRP | 2.483(1.417-4.351) | 0.001 | 3.013(1.841-4.933) | ＜0.001 | 2.692(1.588-4.562) | ＜0.001 |
| Clinical characteristics |  |  |  |  |  |  |
| Age | 0.986(0.959-1.015) | 0.338 | — | — | 0.988(0.961-1.016) | 0.409 |
| Gender | 0.680(0.422-1.096) | 0.113 | — | — | 0.703(0.437-1.129) | 0.145 |
| Tumor location | 0.981(0.753-1.277) | 0.886 | — | — | 0.997(0.768-1.295) | 0.984 |

Model 1: Multivariate analysis including only variables with P < 0.05 in univariate analysis (lnc-IRP and Fe²⁺). Model 2: Fully adjusted multivariate analysis further adjusted for clinical covariates (age, gender, and tumor location). Tumor location was categorized into four subgroups: right-sided colon, left-sided colon, rectosigmoid junction, and rectum. HR, hazard ratio; CI, confidence interval.

Forest plot of multivariate Cox regression analysis (Model 2) for PFS. The vertical dashed line indicates HR = 1. HR > 1 indicates an increased risk of disease progression; HR < 1 indicates a decreased risk.
